# Characterization of Granulations of Calcium and Apatite in Serum as Pleomorphic Mineralo-Protein Complexes and as Precursors of Putative Nanobacteria

**DOI:** 10.1371/journal.pone.0005421

**Published:** 2009-05-01

**Authors:** John D. Young, Jan Martel, David Young, Andrew Young, Chin-Ming Hung, Lena Young, Ying-Jie Chao, James Young, Cheng-Yeu Wu

**Affiliations:** 1 Laboratory of Nanomaterials, Chang Gung University, Gueishan, Taiwan, Republic of China; 2 Adjunct Faculty, Laboratory of Cellular Physiology and Immunology, The Rockefeller University, New York, New York, United States of America; 3 Biochemical Engineering Research Center, Mingchi University of Technology, Taipei, Taiwan, Republic of China; 4 Department of Biochemistry and Molecular Biology, Graduate Institute of Biomedical Sciences, Chang Gung University, Gueishan, Taiwan, Republic of China; 5 Massachusetts Institute of Technology, Cambridge, Massachusetts, United States of America; 6 Harvard University, Cambridge, Massachusetts, United States of America; 7 Research Center of Bacterial Pathogenesis, Chang Gung University, Gueishan, Taiwan, Republic of China; University of California Merced, United States of America

## Abstract

Calcium and apatite granulations are demonstrated here to form in both human and fetal bovine serum in response to the simple addition of either calcium or phosphate, or a combination of both. These granulations are shown to represent precipitating complexes of protein and hydroxyapatite (HAP) that display marked pleomorphism, appearing as round, laminated particles, spindles, and films. These same complexes can be found in normal untreated serum, albeit at much lower amounts, and appear to result from the progressive binding of serum proteins with apatite until reaching saturation, upon which the mineralo-protein complexes precipitate. Chemically and morphologically, these complexes are virtually identical to the so-called nanobacteria (NB) implicated in numerous diseases and considered unusual for their small size, pleomorphism, and the presence of HAP. Like NB, serum granulations can seed particles upon transfer to serum-free medium, and their main protein constituents include albumin, complement components 3 and 4A, fetuin-A, and apolipoproteins A1 and B100, as well as other calcium and apatite binding proteins found in the serum. However, these serum mineralo-protein complexes are formed from the direct chemical binding of inorganic and organic phases, bypassing the need for any biological processes, including the long cultivation in cell culture conditions deemed necessary for the demonstration of NB. Thus, these serum granulations may result from physiologically inherent processes that become amplified with calcium phosphate loading or when subjected to culturing in medium. They may be viewed as simple mineralo-protein complexes formed from the deployment of calcification-inhibitory pathways used by the body to cope with excess calcium phosphate so as to prevent unwarranted calcification. Rather than representing novel pathophysiological mechanisms or exotic lifeforms, these results indicate that the entities described earlier as NB most likely originate from calcium and apatite binding factors in the serum, presumably calcification inhibitors, that upon saturation, form seeds for HAP deposition and growth. These calcium granulations are similar to those found in organisms throughout nature and may represent the products of more general calcium regulation pathways involved in the control of calcium storage, retrieval, tissue deposition, and disposal.

## Introduction

Biomineralization is a general phenomenon in nature that is not restricted to skeletal calcification alone. In fact, ectopic calcification has been generally associated with aging and other pathological processes. Perhaps nowhere else is ectopic calcification more enigmatic and controversial than in the case associated with the so-called nanobacteria (NB). These are slow growing, pleomorphic, sub-micrometer (50–500 nm) entities coated with proteins and containing carbonate hydroxyapatite (HAP) [1]–[8]. NB are supposedly associated with body fluids, blood infusion products, and vaccines and have been linked to numerous diseases involving extra-skeletal calcification as well as various pathologies of infectious nature [1]–[8]. The putative NB found in biological tissues are closely related to the “nannobacteria” found in geological [9], [10] and meteorite [11] samples that have been presented as possible fossilized primitive precursors of biological life. Unique 16S rDNA sequences attributed to NB were ascribed to a new genus termed *Nanobacterium*, which was initially assigned to the α-2 subclass of Proteobacteria [1], [2].

In view of the unusual characteristics of the proposed NB, deemed untenable as seen through the prism of conventional microbiology, the NB phenomenology has been widely contested as lacking evidentiary support [see ref. 12 for an excellent critical review]; [see also ref. 13 for a more comprehensive discussion of related controversies surrounding the NB literature]. In an effort to explain the biology of NB, several studies have presented alternative views [13]–[16]. Cisar et al. [14] showed that NB-like particles could be produced from saliva samples inoculated into fresh medium without however undergoing detectable protein or nucleic acid synthesis expected from the growth of a living entity. Moreover, NB particles could be produced from self-propagating HAP which was shown to nucleate on lipids like phosphatidylinositol [14]. The 16S rDNA sequences previously ascribed to NB were shown to be most likely associated with common environmental microorganisms that are detected as contaminants in PCR [14]. Raoult et al. [15], in turn, described NB as lifeless “mineralo fetuin complexes,” which they termed “nanons.” The main protein was identified as fetuin (referred as fetuin-A in the present study), a result which for the first time links the biology of NB with that of fetuin-A—an association considered paradoxical given that fetuin-A is a calcification inhibitor [15]. “Nanons” were detected with fetuin-A-specific antibodies and were shown to be cytotoxic to several cell types tested [15]. Our own studies [13], [16] showed that serum NB can be explained by self-propagating calcium-protein complexes that convert into HAP in the presence of phosphate. In fact, we also showed that the NB-associated proteins turned out to consist of calcium or apatite binding proteins found in the medium in which NB were presumably formed. In our hands, the main proteins associated with serum NB were shown to include albumin, fetuin-A, apolipoproteins A1 and B100, and complement component 3, which are not only known to have affinities for calcium and apatite, but also are among the most abundant proteins in the serum [13]. As calcification inhibitors, the same proteins that bind avidly to calcium and nascent apatite are seen here to form the scaffolds needed for further nucleation of HAP once they are overwhelmed by the excess of calcium and phosphate ions. This is a dual inhibition-seeding concept that would not only explain the origin of the putative NB, but it also would resolve the paradox of having inhibitors of calcification, like fetuin-A, participate in the formation of the core of NB [13]. However, our own findings called for the potential inclusion of a wider repertoire of calcium and apatite binding proteins, not just fetuin-A, that are all able to induce the inhibition-seeding properties needed to initiate NB-like mineral precipitation [13]. Our studies build on earlier reports [17], [18] and arrives at the conclusion that NB are lifeless, mineralo-organic entities that nonetheless assume morphologies resembling dividing bacteria. This is a peculiar phenomenon of “biomorphic” resemblance that is also found among geological substrates containing calcite, carbonate, and silicate compounds [19]–[22].

Together, these various studies point to the many fallacies inherent in the conclusions drawn from the NB literature, and they question the role proposed for NB in disease pathogenesis. For one, NB can no longer be considered as some novel, primitive, and exotic lifeforms or symbionts, as initially claimed [1], [2]. To date, all NB-proteins identified by us [13], [16] have turned out to consist of common eukaryotic proteins, a result that markedly differs from the earlier findings of prokaryotic proteins, including porins and complex peptidoglycans [2], [23] as well as the bacterial translation elongation factor Tu and the molecular chaperone GroEL [24], all of which had been claimed to be associated with NB. However, in spite of a growing consensus that views NB as lifeless entities—they are now referred also as “calcifying nanoparticles” or CNP—NB continue to be heralded as transmissible and pathogenic agents of an alarming number of diseases that not only include renal and arterial calcifications, but also a variety of other acute and chronic ailments [1]–[8], [25]–[28; see also refs. 12 and 13 for a list of NB–linked diseases]. In this respect, we have found that all NB-like entities incorporate into their scaffold the very serum proteins from the medium used to “support” their culture [13]. In the case of human tissues grown in medium containing fetal bovine serum (FBS), a procedure commonly used by NB proponents to demonstrate the presence of NB in human pathologies, this observation would imply that antigenic epitopes from FBS become incorporated into the NB scaffold. In other words, the putative NB derived from human tissues end up harboring antigens from a different species, creating an unprecedented scenario that compounds the difficulty with drawing any meaningful conclusion regarding the presence of NB in human tissues, not to mention their putative role in pathology.

These studies indicate that the entire NB literature must be reevaluated carefully, and that, furthermore, many of the earlier conclusions drawn on their biology and pathophysiology must be deemed as largely uninterpretable, if not flawed. Nonetheless, it is clear that entities that morphologically and chemically resemble the so-called NB can indeed form from prolonged incubation of cell culture medium supplemented with serum and other body fluids [13]. Armed however with a much better understanding of the structural and chemical characteristics of NB that was advanced through our earlier study [13], along with a growing consensus that these entities represent lifeless mineralo-protein complexes [13]–[18], we are now in a position to look for the source of these particles in the body and to assess whether they can perhaps be found as circulating complexes in the serum. If so, we also seek to determine the condition in which they are present as well as the role, if any, that these particles may have in the body physiology.

A more thorough investigation prompted by these questions is also justified by the fact that calcium granules or inclusion bodies similar to NB are commonly found in nature [see Ryall's excellent and comprehensive review on this subject cited in ref. 29]. In fact, such calcium carbonate or calcium phosphate granules probably represent the most common pathways used for calcium storage, retrieval, and disposal that are found, quoting Ryall [29], “in a diverse range of organisms living in environments as widely apart as water and land” and “spanning an enormous range of phylogenetic complexity.”

In this context, we demonstrate here that mineralo-protein complexes—in the form of laminated granulations, spindles with needle-like projections, and films—are formed directly in both FBS and adult human serum (HS) as a result of calcium or apatite loading and that these complexes resemble the previously described NB as well as calcium deposits and granules seen throughout nature. That is, NB-like precipitating particles will readily form in the serum when this same body fluid is challenged with calcium and/or phosphate ions, a process that does not require the lengthy incubation usually needed to culture NB [1], [2]. Furthermore, discrete proteins bound to these granulations/complexes are found to be indistinguishable from those of NB formed following prolonged incubation in cell cultures. Serum-derived NB may therefore originate from the direct chemical binding of calcium or apatite to calcification inhibitors normally present in the serum that then become seeds for further apatite precipitation and self-aggregation, all of which precludes the need for any proliferation dependent on cell growth or viability. More importantly, these same calcium phosphate complexes or granulations can also be found in normal untreated serum, albeit at much lower amounts, indicating that they may represent physiological products of the normal regulatory pathways that are used to maintain calcium and apatite homeostasis and that somehow become amplified as a result of calcium and/or apatite loading. These same calcium phosphate granulations are most likely disposed from the body, but, presumably, they may also end up participating in either physiological or anomalous calcification reactions. As such, these calcium/apatite granulations may have a much more general role in the body than what had previously been recognized.

## Results and Discussion

### General strategy and experimental outline

Calcification inhibitory factors in the serum with multiple binding sites are conceptualized here to bind avidly to calcium, phosphate, or nascent apatite that upon saturation may precipitate to become seeding templates for further apatite growth. We speculate that the interplay between inhibitory and seeding influences should be physiologically relevant insofar as calcium regulation and homeostasis are concerned. Given that human body fluids are supersaturated with respect to calcium and phosphate ions [30]–[35], it is reasonable to envision that inhibitory forces reigning in their mutual binding and precipitation must be at work in order for these ions to maintain solubility in the body fluids. Likewise, it also follows that upsetting this balance, which would occur when the same inhibitory state is overcome, should result in the precipitation of calcium phosphate and the formation of HAP in a manner and process that are probably repeated throughout nature. This precipitate then needs to be disposed from the body in order to prevent anomalous calcification.

Along this line of reasoning, the same calcium/apatite inhibitory and seeding factors must be readily available in the serum as well as in the other body fluids. According to this simple model, these same calcium-binding inhibitory factors, whether they be fetuin-A, albumin, or other binding sites, should sequester increasing amounts of calcium ions by binding to them until the binding sites for calcium (and/or calcium phosphate) reach saturation. At the point of saturation, the same calcium-inhibitory complexes would then aggregate, become insoluble, and behave as seeds or nidi for further binding of calcium phosphate. This propagation process would continue until microscopic entities similar to the previously described NB and calcium granules commonly found in nature are formed. Assuming this reasoning to be correct, simply adding exogenous calcium or phosphate or a combination of both directly to serum, or perhaps to any body fluid, should produce insoluble mineralo-protein complexes, which when introduced into fresh culture medium should in turn seed even more apatite as well as give rise to the entire NB phenomenology, including the properties of pleomorphism and particle propagation. Extending this same logic, it should also be possible to demonstrate that normal, untreated serum contains these same circulating complexes, albeit at much lower amounts. Structurally and functionally, these various calcium granulations as well as the putative NB should all turn out to be virtually indistinguishable from each other.

### Formation of precipitating complexes in serum following addition of calcium and/or phosphate

Preliminary experiments showed that concentrated solutions of CaCl_2_, Na_2_HPO_4_, or a combination of both, could be added slowly in a drop-wise manner to either filtered FBS or HS, followed by vigorous shaking, without resulting in immediate precipitation. In this manner, CaCl_2_ could be slowly added to FBS to concentrations as high as 50 mM without inducing precipitation even after one hour of incubation at 37°C or after two hours of incubation at room temperature. Likewise, under the same incubation conditions, up to 100 mM of CaCl_2_ could be added to several batches of HS without resulting in any precipitation. The lack of precipitation in any one of these experiments could be ascertained by centrifugation at 16,000×*g* at room temperature for 1 hour, which would have been expected to sediment NB-like complexes [13]. In a similar manner, Na_2_HPO_4_ could be added up to 30 mM to several lots of serum without any apparent precipitation. In marked contrast, when both CaCl_2_ and Na_2_HPO_4_ were added together to either FBS or HS, precipitations appeared readily when as low as 3 mM of each ion were introduced, indicating that the propensity for precipitation greatly increased when both ions were added simultaneously.

On the other hand, these same preliminary experiments also revealed a marked variability of results depending on the lot of serum used. That is, different batches of FBS and HS produced significant differences in the extent of ion-triggered precipitation. Significant differences were also noticed depending on the age of the serum lot tested. Compared to fresh serum for example, aged serum (e.g., serum that had been stored for several weeks) tended to produce precipitates more readily when the same amounts of calcium and/or phosphate were added. As a result of aging, some batches of serum were seen to produce precipitates spontaneously through storage alone and without any ion treatment, but in general untreated serum produced little or no visible precipitation upon centrifugation.

The same experiments also revealed marked differences in precipitation depending on the length of incubation allowed following the addition of CaCl_2_ and Na_2_HPO_4_ (for brevity, these will be referred respectively as calcium and phosphate treatments in the text). Thus, while short incubations as described above did not lead to any visible precipitation when individual ions were added even to artificially high concentrations, prolonging the incubation after the same serum treatment produced steady precipitation as a function of time. In fact, either calcium or phosphate produced precipitation when added to 6 mM or 3 mM, respectively, provided that the subsequent incubation was prolonged to 2 hours at 37°C or overnight at room temperature. Likewise, under these incubation conditions, precipitation was observed when both calcium and phosphate were added together to as little as 1 mM each. With longer periods of incubation, the precipitating concentrations of the same ions could be further reduced. For some serum batches, precipitation was noticed with as little as 0.1 mM calcium phosphate added when the subsequent incubation was lengthened to 48–72 hours at 37°C.

### Precipitating calcium and apatite complexes prepared in serum can seed self-propagating particles resembling NB

In all instances of serum treatment with precipitating ions, white, granular precipitates that could be dispersed in solution became visible. In the presence of excess calcium, pellets appeared granular and fluffy to the eye, while the excess of phosphate produced gelatinous, grayish pellets that appeared more clumpy and resistant to dispersion even by vigorous shaking. These visual characteristics could be easily modulated by adding different amounts of calcium and phosphate.

**Figure 1:**
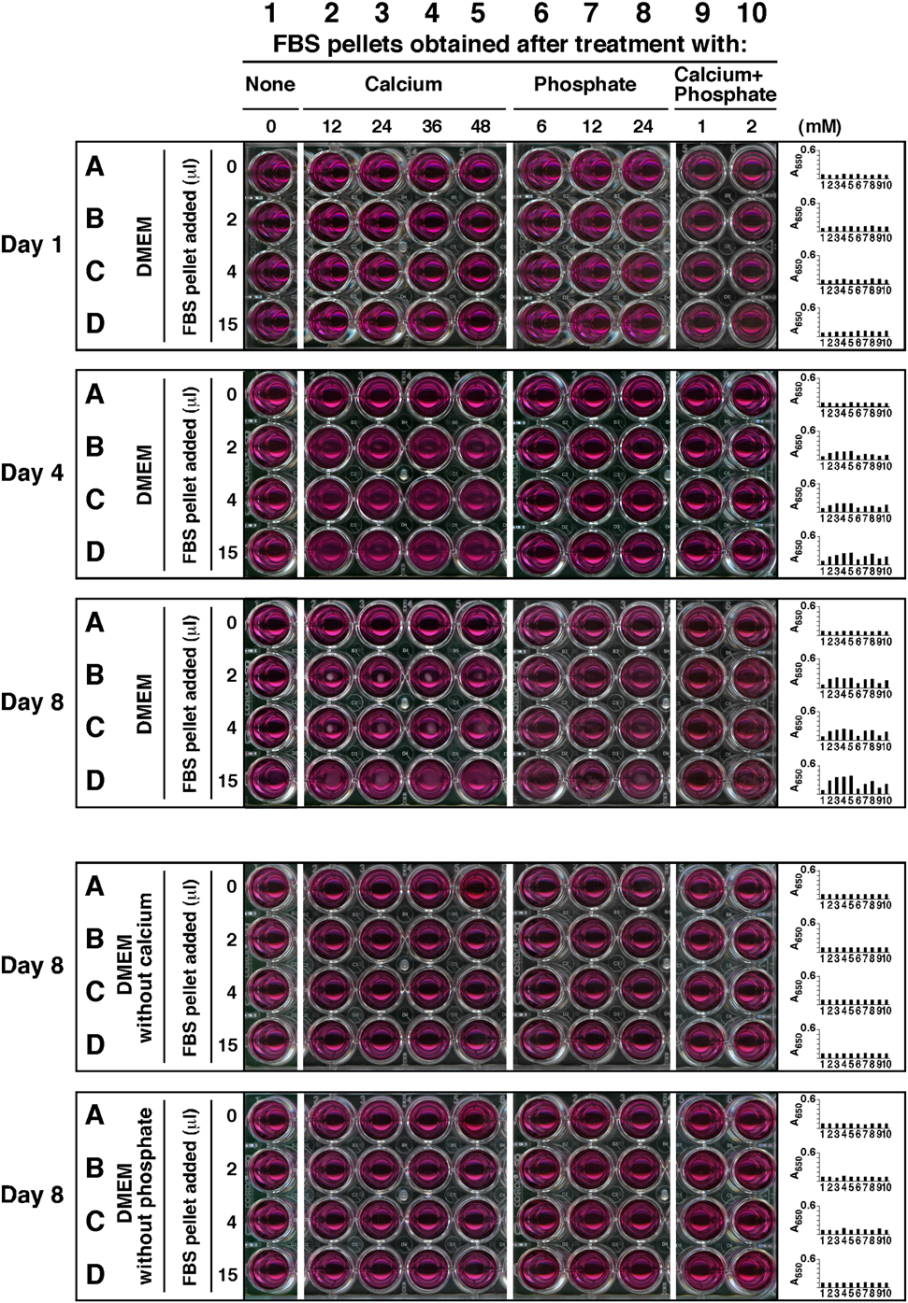
Calcium and phosphate added to serum produce particle-seeding pellets. Serum pellets were obtained by adding calcium and/or phosphate to FBS to the amounts indicated at the top and incubated overnight at room temperature. Treatment of serum was divided into 4 groups of ions added: no exogenous ions (column 1, “None”); CaCl_2_ (columns 2–5, referred as “Calcium,” 12–48 mM); Na_2_HPO_4_ (columns 6–8, “Phosphate,” 6–24 mM); and a combination of both CaCl_2_ and Na_2_HPO_4_ (columns 9 and 10, “Calcium+Phosphate,” at either 1 mM or 2 mM each). After overnight incubation, the pellets were obtained and processed as described in the *Materials and Methods*. The resuspended pellets were inoculated into either serum-free DMEM (rows A–D, top 3 panels), calcium-free DMEM (rows A–D, 4^th^ panel from top), or phosphate-free DMEM (rows A–D, bottom panel) in the same order, as three exact replicas. The amount of resuspended pellet volume inoculated into each well is depicted on the left. Observations were done on days 1, 4, and 8 for the top three panels and on day 8 for the bottom two. All incubations were done at 37°C in cell culture conditions. The wells in column 1 corresponded to inoculation with serum pellets obtained from FBS without any ion treatment, while the wells in row A represented control DMEM, without any pellet inoculation. These controls showed no turbidity by visual inspection. On the other hand, turbidity and the presence of precipitates could be seen in all the other wells of the “Day 4” and “Day 8” panels. The wells of the 4^th^ panel, corresponding to incubation in DMEM without calcium, did not show any precipitate whereas the bottom panel wells (DMEM without phosphate) produced small amounts of precipitates.

Next, for purposes of seeing whether these same pellets were capable of seeding NB-like particles, they were collected by centrifugation, washed, and serially transferred into fresh, new medium. Figure 1 illustrates dose-dependent experiments in which FBS pellets were first obtained after serum treatment with the indicated amounts of ions, and then inoculated into serum-free Dulbecco's Modified Eagle's Medium (DMEM). Inoculation of the serum pellets into DMEM did not produce any turbidity initially while A_650_ reading remained low (Fig. 1, top “Day 1” panel, rows A–D, wells 2–10, corresponding to the first day of inoculation), but following incubation at 37°C in cell culture conditions, pellets inoculated into serum-free DMEM developed precipitates steadily over time in a dose-dependent manner that became noticeable to the naked eye or through A_650_ reading by the third day of incubation and that increased thereafter. In Figure 1, data collected on days 4 and 8 are shown to illustrate a time-dependent as well as dose-dependent increase in the amount of precipitation formed following the initial inoculation (Fig. 1, “Day 4” and top “Day 8” panels, rows A–D, wells 2–10). The amount of precipitation increased less after the first week, and by the end of the second week, it was seen to plateau (not shown). From these data, it is clear that serum pellets obtained and processed in this manner displayed clear particle-seeding activity that could not be attributed solely to the initial inocula since the turbidity was seen to increase over time.

In the case of calcium, a clear dose-dependent effect could be seen when calcium was added to FBS to a final concentration between 12 and 48 mM, followed by inoculation of the obtained pellets into serum-free DMEM and incubation for several days (Fig. 1, “Day 4” and top “Day 8” panels, rows A–D, wells 2–5). This dose-dependent effect was obvious depending on the amount of calcium initially added to the serum, with more calcium conferring greater seeding potential (in Fig. 1, “Day 4” and top “Day 8” panels, rows A–D, compare wells 2–5; note the dose-dependent effect going from left to right). Furthermore, for each concentration of calcium initially added to induce pellet formation, a dose-dependent effect could also be seen for the amount of FBS-pellet used for inoculation into serum-free DMEM, with larger serum pellets inducing larger NB-like precipitates (Fig. 1, “Day 4” and top “Day 8” panels; note a dose-dependent effect within the same panel going from top to bottom, that is, from wells B to D, corresponding to the inoculation of 2 µl to 15 µl of FBS-pellet, respectively). Similar results were obtained with HS pellets (not shown).

Alternatively, serum pellets could be serially transferred to new medium at 1∶5 dilution for at least 5 cycles, indicating a self-propagating nature similar to what had been reported earlier for NB [1]–[8]. However, the precipitates were seen to decrease in size with each transfer into serum-free DMEM; a similar decrease in precipitate volume was also seen with the serial transfer of several strains of NB into serum-free medium (see *Materials and Methods*; data not shown).

FBS that had been treated with phosphate alone also produced pellets (Fig. 1, “Day 4” and top “Day 8” panels, with wells marked by columns 6–8 corresponding to a range of 6–24 mM of phosphate, respectively). Like calcium, phosphate also produced similar dose-dependent effects (compare wells in columns 6–8 with columns 2–5, Fig. 1, “Day 4” and top “Day 8” panels, with an increase in precipitation from columns 6 to 8; observe also the increase in precipitation within the same panel from wells 6B to 6D; 7B to 7D, and 8B to 8D, that is, within each column).

As noted earlier, serum pellets were more effectively produced by a combination of calcium and phosphate added together. In fact, FBS-pellets produced by the addition of calcium and phosphate at the relatively low concentration of 1 mM each, followed by a 2-hour or overnight incubation, displayed clear particle-seeding activity when subsequently inoculated into serum-free DMEM (Fig. 1, “Day 4” and top “Day 8” panels; compare columns 9 and 10 within the same panel; compare also for example well 10B with wells 10C and 10D within the same panel). As before, a distinct dose-dependence could be discerned with the amount of calcium phosphate added initially to obtain the serum pellet as well as with the final pellet volume used for inoculation into DMEM (Fig. 1, “Day 4” and top “Day 8” panels; compare rows B, C, and D for each column as well as among columns 9 and 10 within the same panel). The mineral pellets initially tended to aggregate at the center of each well until they dispersed to resemble membrane-like formations. Similar results were also obtained with HS-pellets (data not shown).

The experiments shown in Figure 1, done with excess calcium or phosphate added to serum, demonstrate the marked capacity of both FBS and HS to absorb exogenously added ions, resulting in little or no precipitation. However, upon prolonged incubation of calcium- or phosphate-treated serum at 37°C for a period of several days or weeks, we noticed precipitation with much lower amounts of either ion added (1–10 mM each of either calcium or phosphate and 0.1–1 mM of each with calcium and phosphate combined). The pellets obtained under these conditions also showed clear particle-seeding activity. Likewise, increasing the speed of centrifugation to 140,000×*g* for 1 hour significantly increased the size of the pellet obtained (data not shown), indicating that some of the complexes may have remained in suspension in the ion-treated serum following the lower-speed centrifugation (16,000×*g*) used before.

None of the control wells containing only DMEM and devoid of any serum-derived materials produced any significant amounts of precipitates, as discerned visually or through A_650_ reading following the same 3-day incubation (Fig. 1, in each one of the 5 panels). Significantly, control FBS that had not received exogenous calcium or phosphate produced a small precipitate after centrifugation, but this material did not lead to the nucleation of any significant amount of precipitates during the same 4-day period of observation under the conditions used (see Fig. 1, column 1 of the “Day 4” panel) or even after prolonged incubation for 1 week (Fig. 1, column 1 of the top “Day 8” panel). This important negative control indicated that the seeding factor seen here could not be attributed solely to any insoluble matter or debris present in FBS prior to the addition of calcium and/or phosphate ions. In this regard, it should be noted that our initial double filtration of serum through both 0.2 m and 0.1 m membranes was intended to remove as much insoluble material as possible from the FBS lots used in this study. It should be pointed out however that the negative results obtained with untreated serum pellets were not absolute since larger amounts of this untreated serum pellet did indeed produce NB-like precipitates upon subsequent inoculation into DMEM (not shown); likewise, upon longer incubations up to 1 month in DMEM, precipitates could be detected, albeit at much lower levels when compared with the other serum pellets mentioned earlier (not shown). Again, the various lots of serum produced widely different amounts of pellet upon centrifugation. Moreover, by increasing the speed of centrifugation to 140,000×*g* or by storing the serum batch for several weeks at 4°C, a significantly larger amount of pellet could be obtained from untreated serum, suggesting again that circulating calcium-phosphate-complexes in the serum, if any, may remain initially in solution until heavier loads of calcium and phosphate are added that result in binding, saturation, and precipitation. It is also obvious that this same propensity for precipitation can be induced in the case of untreated control serum either by higher speed of centrifugation or by prolonged storage conditions. Together, these results suggest that while adding exogenous calcium and phosphate to serum results in the formation of precipitating complexes, this same process may occur in control, untreated serum as well, albeit at much slower rates.

An additional control was done to assess whether the NB seeding activity observed here with serum pellets is dependent on the presence of both calcium and phosphate present in the inoculated medium. As shown in the two bottom panels of Figure 1, we inoculated the various serum pellets into DMEM that lacked either calcium or phosphate. No significant amount of precipitate was generated under these conditions even after several days of incubation in cell culture conditions. Together, these experiments reveal that serum can sequester excess calcium and phosphate until precipitating complexes are formed, which in turn seed further particle growth only in the presence of both calcium and phosphate in the surrounding milieu. Our experiments also show that either calcium or phosphate added alone to serum is sufficient to generate precipitation, but only when the ions are added to artificially high concentrations. The high absorption capacities for calcium or phosphate displayed by serum attests to the efficacy of the built-in inhibitory mechanisms used to prevent calcification. On the other hand, when added simultaneously to serum, calcium and phosphate trigger precipitation at much lower ion levels, indicating that the same inherent calcification-inhibitory mechanisms are much more easily overcome when both precipitating ions are present.

### Precipitating serum complexes appear as calcium and apatite granulations that resemble morphologically both bovine and human NB

**Figure 2:**
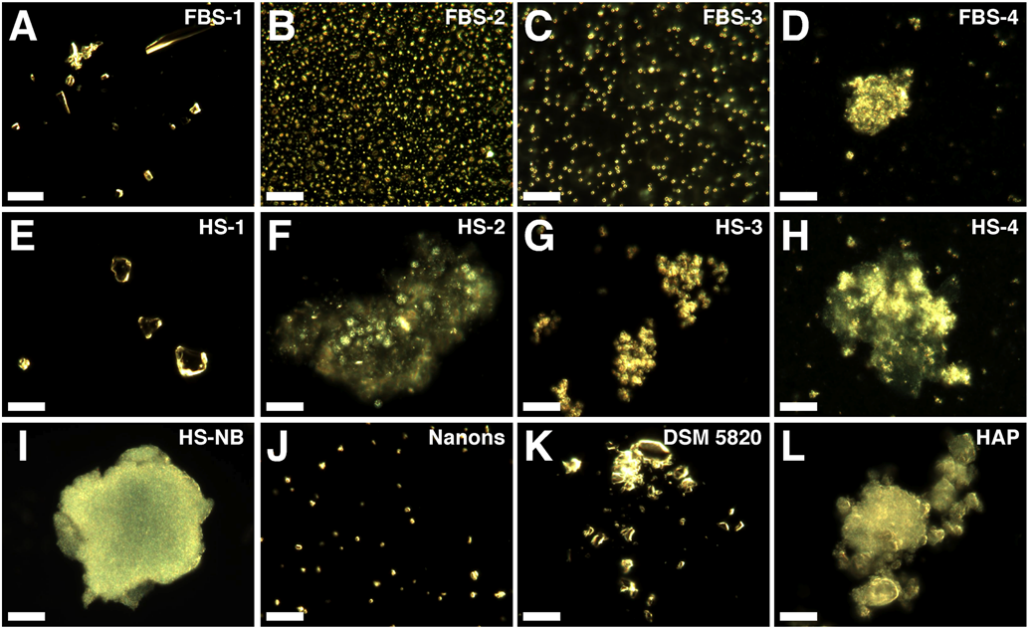
Serum pellets and NB have similar pleomorphic morphologies under dark field microscopy. Serum pellets were prepared from untreated serum (A and E) or following addition of either 48 mM CaCl_2_ (B and F), 24 mM Na_2_HPO_4_ (C and G), or a combination of both 2 mM CaCl_2_ and 2 mM Na_2_HPO_4_ (D and H) to the indicated serum, followed by incubation at room temperature overnight, centrifugation, and washing steps as described in the *Materials and Methods*. Low amounts of large heterogeneous particles were noted in the untreated serum pellets (A and E) while the other serum pellets (B–D, F–H) produced smaller and more homogeneous round particles. Such round particles tended to aggregate forming clumps and granular patches (D, F–H). Note that individual granules can be discerned against a background of aggregated material. Similar morphologies were noted in the controls, with HS-NB (I) showing an aggregated diffuse mass in which granules are embedded; “nanons” (J) as dispersed round particles; and DSM 5820 (K) as clumps of highly refringent particles. (L) HAP is shown for comparison. Scale bars: 10 µm.

**Figure 3:**
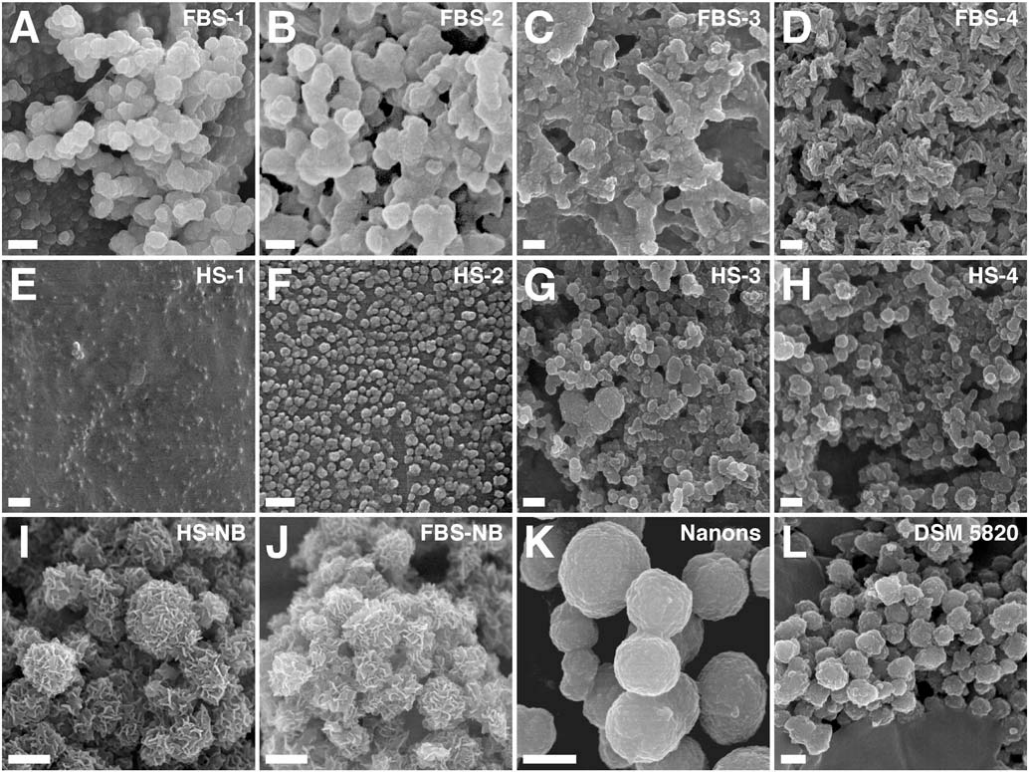
Morphology of serum pellet granulations seen under SEM demonstrates resemblance to NB. Serum pellets were prepared as in Fig. 2 from untreated serum (A and E) or after addition of either CaCl_2_ (B and F), Na_2_HPO_4_ (C and G), or a combination of both CaCl_2_ and Na_2_HPO_4_ (D and H) to the indicated serum (amounts of ions added as described in *Materials and Methods*). Serum pellets primarily harbored round particles in FBS pellets (A–C) and these particles tended to be smaller in HS (E–G). Treatment with both CaCl_2_ and Na_2_HPO_4_ produced particles undergoing different stages of crystallization and film coalescence as well as needle-like projections or spindles (D and H). The morphologies of the serum pellets were similar to the NB controls obtained from either 10% HS (I) or 10% FBS (J) as well as to the NB strains “nanons” (K) and DSM 5820 (L), even though the sizes of the serum pellets particles tended to be smaller than NB. Note that while both “nanons” and DSM 5820 showed smooth surfaces, HS-NB and FBS-NB appeared with rough surfaces containing needle-like crystalline projections. Scale bars: 100 nm (B); 250 nm (C–E, G, H); 300 nm (F, L); 500 nm (I, J); 600 nm (K); 1 µm (A).

**Figure 4 pone-0005421-g004:**
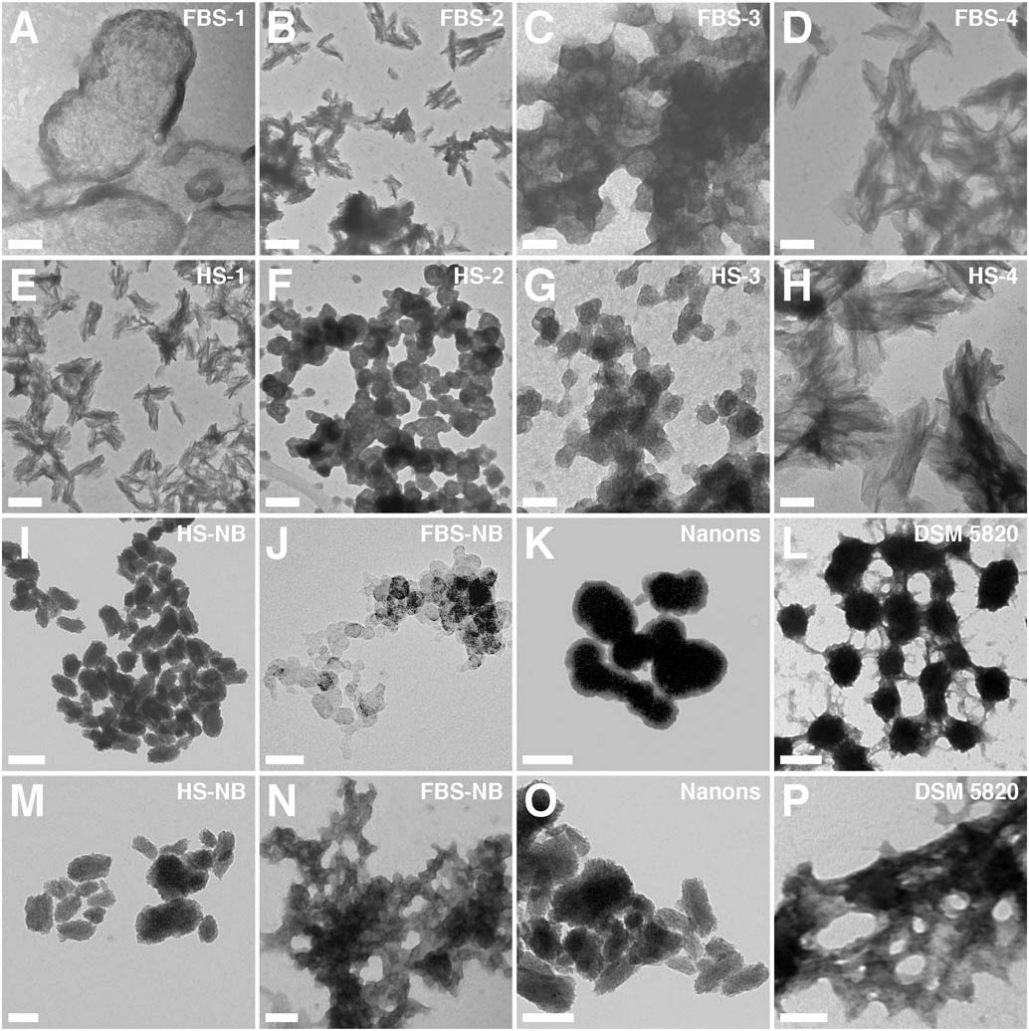
Morphology of unstained serum pellet particles observed by TEM shows similarity to NB. Serum pellets were prepared from untreated serum (A and E) or after addition of either CaCl_2_ (B and F), Na_2_HPO_4_ (C and G), or both (D and H) to the indicated serum, followed by preparation for TEM. Samples were viewed without fixation or staining. The serum pellets displayed various morphologies including round particles (A, C, F, and G) while other samples harbored spindles with more crystalline appearance (B, D, E, and H). In general, the combination of calcium and phosphate tended to produce more readily spindles with needle-like crystalline projections (D and H). The various NB controls shown in the bottom two rows were displayed to show comparable morphologies with predominant round particle shapes (3^rd^ row) or more crystalline aggregates (4^th^ row). However, morphological variations can still be seen within each row. Thus, NB cultured directly from 10% HS (I) or 10% FBS (J) displayed predominantly chain-linked ovoid or spherical shapes resembling dividing bacteria. In contrast, the NB strains “nanons”(K) and DSM 5820 (L) shown here appear further along in their crystallization and while they have retained ovoid shapes, they also show more pronounced fusion and aggregation. In the case of “nanons” (K), there are noticeable thick walls that appear less electron-dense than the core, presumably formed of minerals. For DSM 5820 (L), crystalline bridges can be seen connecting the fused ovoid particles. (M–P) show further progression in aggregation and crystallization, with the appearance of coalesced films (more noticeable in N and P). Film-like aggregation is generally seen with longer periods of incubation or by reducing the amount of serum in the culture medium (to less than 3%). Scale bars: 50 nm (C, G, H, J); 100 nm (A, D, F, N, P); 200 nm (B, E, I, K–M, O).

**Figure 5:**
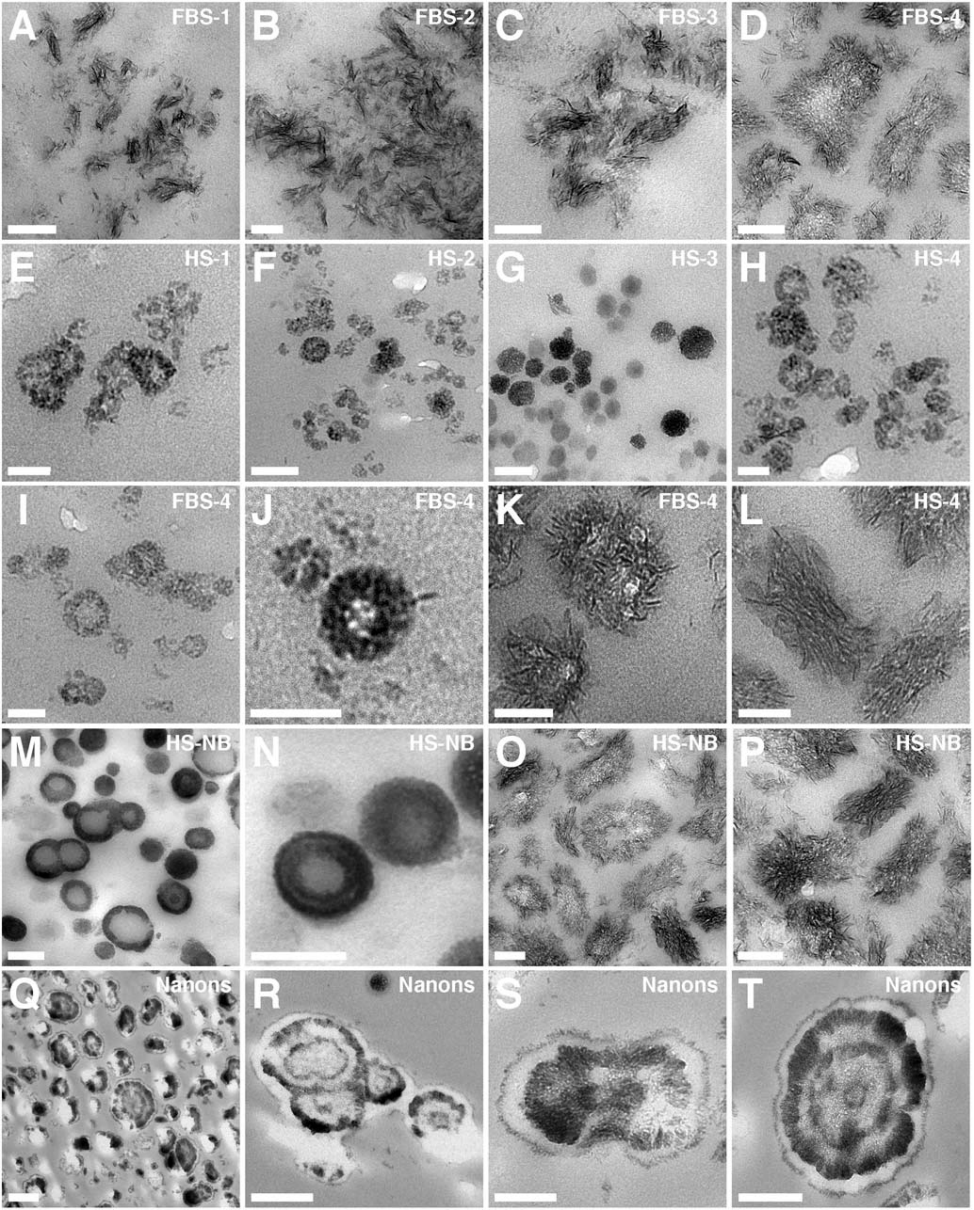
Morphological resemblance between serum pellets and NB as seen by thin section TEM. Serum pellets obtained from untreated serum (A and E) or after addition of either CaCl_2_ (B and F), Na_2_HPO_4_ (C and G), or a combination of both (D, H–L) to the indicated serum, were processed for thin section TEM as described in the *Materials and Methods*. The FBS pellets (A–D) show spindles in various stages of crystallization and aggregation, with (A and B) consisting of smaller filamentous stacks while (C and D) show spindles in the process of fusion and coalescence, presumably to form crystallized films. On the other hand, the HS granulations (E–H) show predominantly round particles with a diameter between 20 to 100 nm. (I–L) represent magnified images of serum granulation specimens to illustrate marked pleomorphism and heterogeneity within the samples prepared in FBS (I–K) or HS (L). (I and J) show FBS-4 round particles appearing with thick, single walls, which appear to represent precursors to the larger fused ovoid particles containing crystallized needle-like projections (K). These appear to coalesce further until they form the spindles appearing as stacks of crystalline filaments arranged co-linearly, shown here for HS-4 (L); note that the various HS samples appear predominantly as round shapes, thus this image was shown to emphasize the phenomenon of pleomorphism. Large structures with ring-like formations were characteristic of NB cultured directly from 10% HS (M–P) and “nanons” cultured in 10% FBS (Q–T). In (S), “nanons” can be seen to be fusing to form stacks of filaments. Note the presence of spherical shapes with two (Q), three (R and S), and four (T) concentric rings of electron-dense material. In general, “nanons” (Q–T) tended to be larger than the similar formations seen in the serum pellets (K and L). Scale bars: 50 nm (E, I–L); 100 nm (B–D, F–H, O, P), 200 nm (A, M, N, S); 300 nm (T); 500 nm (Q, R).

**Figure 6 pone-0005421-g006:**
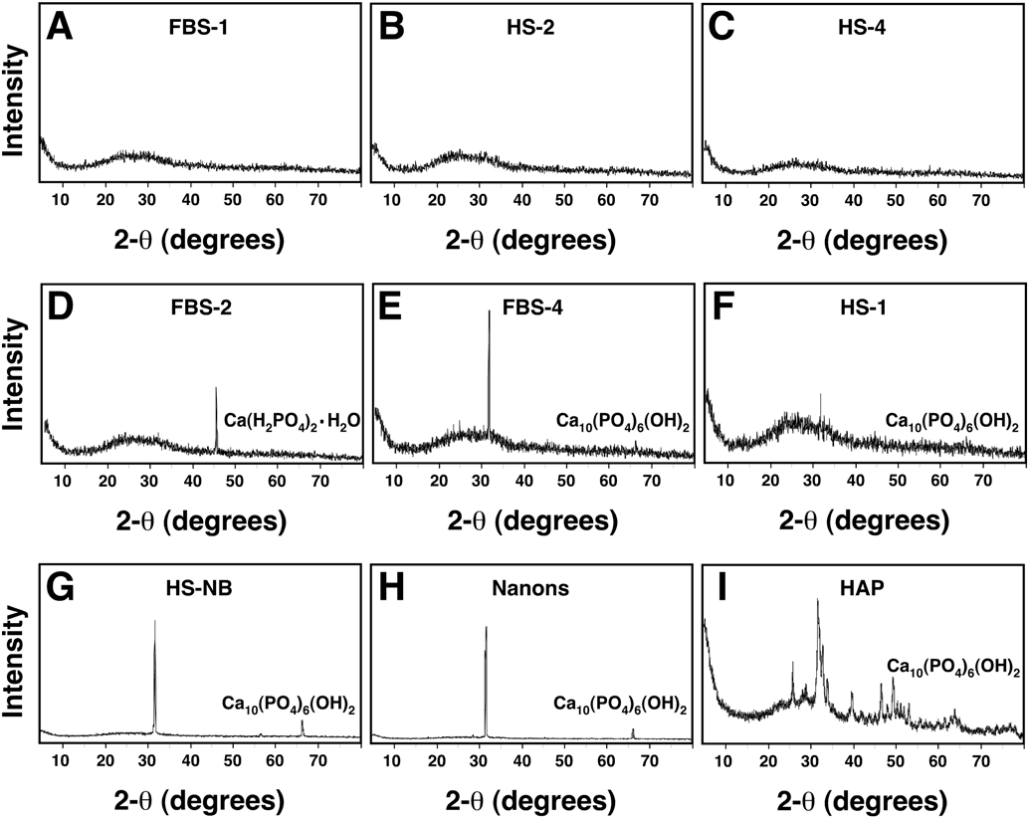
Powder X-ray diffraction spectra of serum granulations demonstrating both amorphous and crystalline patterns. Serum granulations (pellets) were obtained from either untreated serum (A and F) or following treatments with CaCl_2_ (B and D) or a combination of CaCl_2_ and Na_2_HPO_4_ (C and E) to the indicated serum, and were dried prior to XRD analysis. Note that the XRD spectra include amorphous patterns (A–C) and peaks corresponding to crystalline compounds of Ca(H_2_PO_4_)_2_·H_2_O (D) and Ca_10_(PO_4_)_6_(OH)_2_ (E and F). Crystalline patterns were seen associated with both calcium (D) and calcium phosphate-treated sera (E) as well as untreated serum (F), whereas amorphous patterns were seen not only with untreated serum (A), but also with calcium-treated (B) and calcium phosphate-treated sera (C). XRD spectra corresponding to Ca_10_(PO_4_)_6_(OH)_2_ were obtained for NB cultured from 10% HS (G) or 10% FBS (H) as well as commercially available HAP used for comparison.

**Figure 7 pone-0005421-g007:**
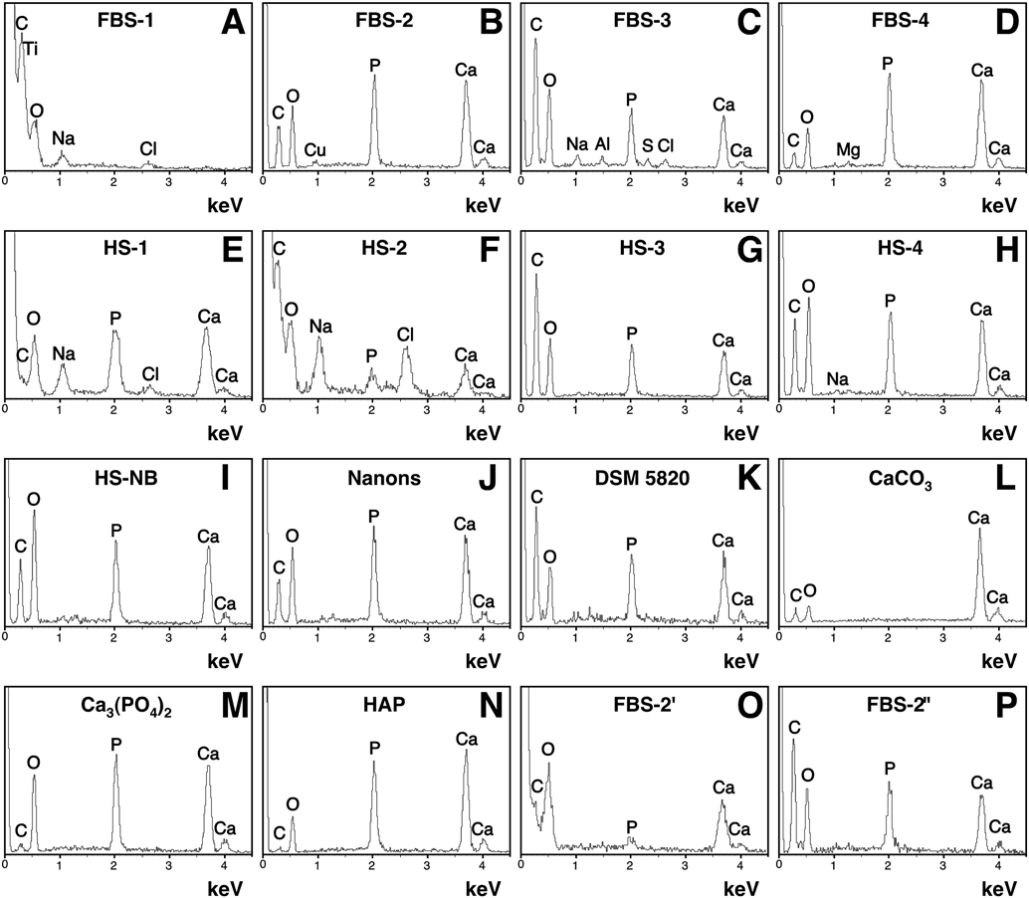
Energy-dispersive X-ray spectroscopy of the serum granulations reveals a broad spectrum of compositions similar to NB. Serum granulations (pellets) were obtained as before from untreated serum (A and E) or after addition of either CaCl_2_ (B and F), Na_2_HPO_4_ (C and G), or a combination of both (D and H) to the indicated serum. EDX spectra were also obtained for NB cultured from HS (I) or for two NB strains cultured in FBS (J and K). Commercial reagents of CaCO_3_ (L), calcium phosphate labeled as Ca_3_(PO_4_)_2_ (M), and HAP (N) were used for comparisons. Serum granulations were also prepared by adding CaCl_2_ to FBS followed by incubation for 1 hour (O) or overnight (P) at room temperature. The following Ca/P ratios were obtained: (B) 1.31; (C–E) 1.33; (F) 1.86; (G) 1.3; (H) 1.24; (I) 1.28; (J) 1.36; (K) 1.31; (M) 1.31; (N) 1.63; (O) 5.42; and (P) 1.24. In (A) and (L), Ca and P were not detected. The presence of C in some samples might be attributed in part to the formvar carbon-coated grids used as support for the analysis.

**Figure 8 pone-0005421-g008:**
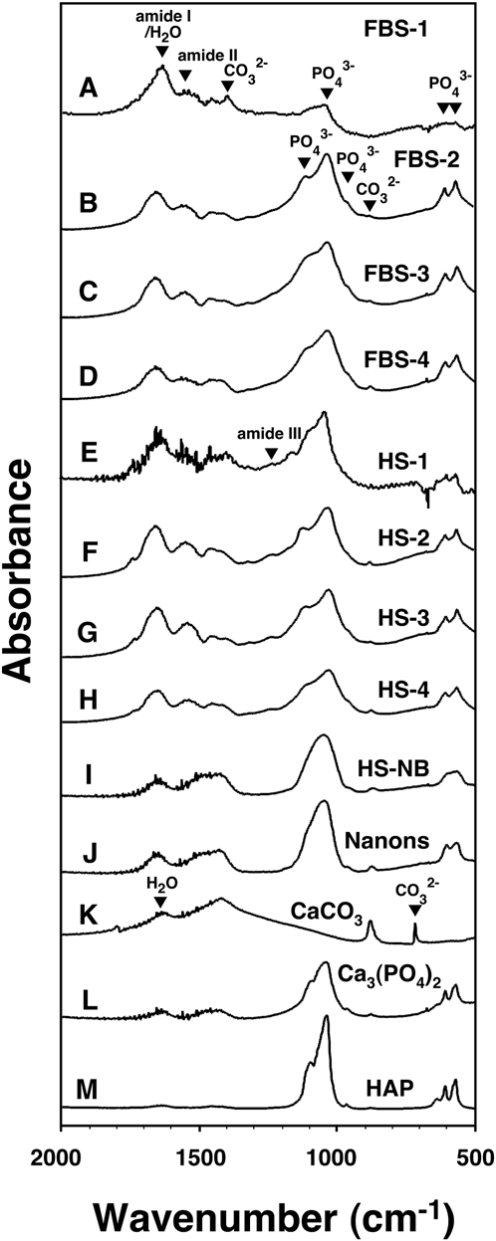
Fourier-transformed infrared spectroscopy of serum granulations reveals the presence of carbonate and phosphate. Serum granulations (pellets) were prepared from untreated serum (A and E) or following addition of either CaCl_2_ (B and F), Na_2_HPO_4_ (C and G), or a combination of both (D and H) to the indicated serum. The FTIR spectra of the serum granulations revealed peaks characteristic of phosphate at 575 cm^−1^, 605 cm^−1^, 960 cm^−1^, and 1,000–1,150 cm^−1^ as well as carbonate at 875 cm^−1^ and 1,400–1,430 cm^−1^. Peaks corresponding to amide I, II, and III near 1,660 cm^−1^, 1,550 cm^−1^, and 1,250 cm^−1^, respectively, were also noted in the serum granulations (A–H) probably corresponding to the presence of proteins from the serum used. NB cultured from 10% healthy HS incubated in DMEM for one month (I) and the NB strain “nanons” cultured in 10% FBS (J) showed FTIR spectra identical to those of serum granulations except for the amide peaks which were of lower intensity for amide I and were absent for amide II. Note that the presence of residual water seen in the controls (K–M) at 1,650 cm^−1^ could also contribute to the intensity of the peak corresponding to amide I. Commercially available CaCO_3_ (K), Ca_3_(PO_4_)_2_ (L), and HAP (M) were included for comparison.

**Figure 9 pone-0005421-g009:**
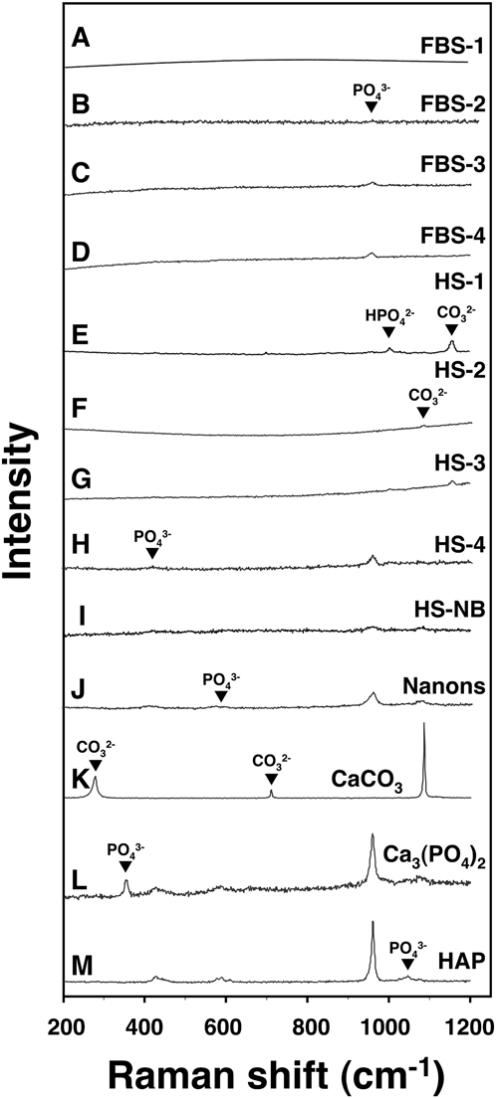
Micro-Raman spectroscopy shows similar chemical compositions for the serum granulations and NB. Serum granulations (pellets) obtained from untreated serum (A and E) or following addition of either CaCl_2_ (B and F), Na_2_HPO_4_ (C and G), or a combination of both (D and H) to the indicated serum, were submitted to micron-Raman spectroscopy. NB cultured from 10% healthy HS in DMEM (I) and the NB strain “nanons” cultured in 10% FBS (J) were also included for comparison. The various serum granulation/pellet and NB specimens showed marked variability of peaks with phosphate ions being obtained more consistently. Phosphate peaks were seen at 361 cm^−1^, 440 cm^−1^, 581 cm^−1^, 962 cm^−1^, 1,002 cm^−1^ (HPO_4_^2−^), and 1,048 cm^−1^ whereas carbonate peaks were noticed at 280 cm^−1^, 712 cm^−1^, 1,080 cm^−1^, and 1,150 cm^−1^. Commercially available CaCO_3_ (K), Ca_3_(PO_4_)_2_ (L), and HAP (M) were included for comparison.

**Figure 10 pone-0005421-g010:**
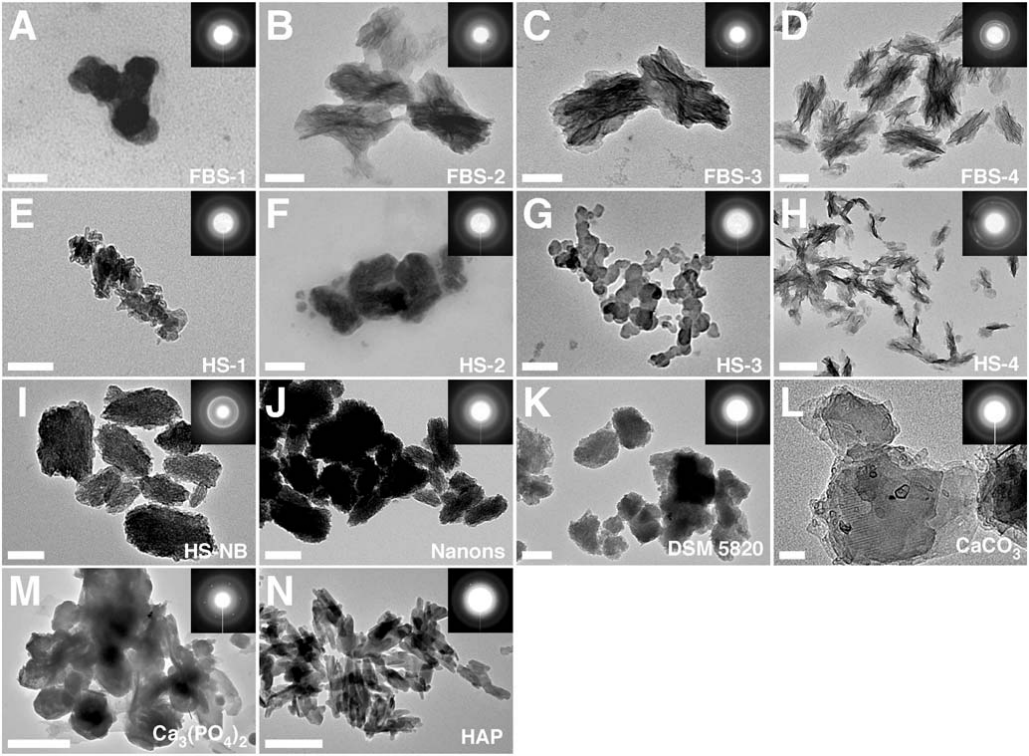
Serum granulations and NB specimens show similar electron diffraction patterns. Serum granulations (pellets) obtained from untreated serum (A and E) or following addition of either CaCl_2_ (B and F), Na_2_HPO_4_ (C and G), or a combination of both (D and H) to the indicated serum, were processed for TEM without fixation or staining. NB cultured from 10% HS (I) as well as the NB strains “nanons” (J) and DSM 5820 (K) cultured in 10% FBS were also included for comparison. The electron diffraction patterns shown in the insets indicated that the serum granulations and the NB samples consisted of polycrystalline minerals either with a low degree of crystallinity as shown by the fuzzy rings (A, B, E–G) or with a more crystalline mineral phase as shown by the presence of arrays of dots on the corresponding diffraction patterns (C, D, H). In comparison, the commercial reagents CaCO_3_ (L), Ca_3_(PO_4_)_2_ (M), and HAP (N), used as controls, more consistently displayed a high degree of crystallinity as shown by the presence of dots on their electron diffraction patterns. Scale bars: 50 nm (A, L); 100 nm (B–E, G, I, J); 200 nm (F, H, K, N); 500 nm (M).

We next examined the morphology of serum pellets obtained through the addition of either calcium or phosphate, or a combination of these two ions. Various low and high resolution microscopies were used. For easier referencing of the individual images within the figure composites shown as Figures 2, 3, 4, 5, 6, 7, 8, 9,10 and for purposes of abbreviation, we adopted the following nomenclature. In addition to the identifying letters used for each figure, all figures were also labeled as either FBS- or HS- followed by a number code, explained as follows. FBS-1/HS-1 represented serum pellets from FBS or HS, respectively, obtained under untreated conditions and used as controls; FBS-2/HS-2 corresponded to the addition of calcium alone to the serum sample prior to incubation and centrifugation; FBS-3/HS-3 corresponded to the addition of phosphate alone; and FBS-4/HS-4 corresponded to the addition of both calcium and phosphate.

By dark field microscopy and in the absence of ion treatment, both FBS and HS pellets (FBS-1 and HS-1) produced little detectable material that appeared as highly refringent and heterogeneous particles as well as patches varying in size markedly between 0.1 to 10 µm (Fig. 2A and E, respectively). This strong refringence as seen by dark field microscopy is probably due to the optically dense walls of these particles, which also make the same particles easily discernible by phase contrast microscopy in the absence of any staining (not shown).

Upon addition of calcium or phosphate to serum, however, pellets showed a large number of smaller and more homogeneous particles with sizes typically under 200 nm (Fig. 2B and C). On higher magnification under a dark field microscope, the particles appeared to display a refringent membrane-like contour (not shown). Upon addition of both calcium and phosphate, the particles appeared to aggregate, forming large patches within which the individual spheroid particles could still be discerned (Fig. 2D and H). The granular patch or film was not restricted only to treatment with both calcium and phosphate; serum treated with either calcium or phosphate alone generated pellets that eventually converted to this same morphology. For purposes of illustration, both Figure 2F and G clearly display the granular-patch morphology. With prolonged storage for 1–2 weeks, even at 4°C, we noticed that all pellet samples appeared to transition from the small spherical particle shapes to the granular membranous patches, and these in turn appeared to aggregate further until only the larger clumps of refringent material were left, at the expense of the individual spherical shapes, which were eventually lost (not shown). Compared to the calcium-phosphate-treated serum pellets, the control untreated pellets gave comparable dark field images except for the much smaller amounts of precipitate material seen (Fig. 2A and E).

These images were remarkably similar to those obtained from previously characterized NB and chosen as controls in our study (Fig. 2I–K). To make sure that our controls were indeed representative of NB reported in the literature, we took into account first that all NB described to date had been derived from cell cultures initially containing serum, usually FBS or HS at 10%, that were then subcultured through serial passage (referred here as either FBS-NB or HS-NB, abbreviated respectively from the more precise terms FBS-derived NB or HS-derived NB) [1], [2]. Figure 2I shows an example of HS-NB that had been cultured in DMEM inoculated with 10% HS collected from a healthy donor, exactly as described in the protocols published earlier [1], [2] and outlined in the *Materials and Methods*. In addition, we used the earlier described “NB strains” derived from cell culture medium inoculated with 10% FBS, which included three strains of putative *Nanobacterium* sp. that were deposited by Kajander with the German Collection of Microorganisms (referred by their serial codes DSM 5819–5821) [23], [36] as well as the *Nanobacterium* sp. strain Seralab 901045 used by Raoult et al. [15], which they have called “nanons” (see Fig. 2J for “nanons” and Fig. 2K for one of the three DSM strains). We reasoned that these controls would allow comparison of our data with both bovine and human NB, while the use of previously characterized NB “strains” would remove any doubt concerning their origin or representation. As can be seen in Figure 2I–K, an array of morphologies could be discerned again that included spherical particles (shown for “nanons,” Fig. 2J), granular patches showing the spherical aggregated particles (DSM 5820, Fig. 2K), and clumps of smooth refringent material no longer containing any evidence of round particles (HS-NB, Fig. 2I). These three broad stages of morphological development were identical to those seen with the serum pellet particles. As an additional control, chemically synthesized HAP was also photographed to show its typical monolithic solid morphology (Fig. 2L).

Serum pellets were next studied by higher resolution imaging techniques like scanning electron microscopy (SEM) and transmission electron microscopy (TEM) (Figs. 3–5). As seen by both SEM (Fig. 3) and TEM (Figs. 4 and 5), these pellets gave a wide spectrum of morphologies, ranging from round, 20–200 nm particles to coalesced mattresses or films. On average, the size of the round particles seen in the HS pellets (at 20–150 nm) was smaller than those seen in pellets prepared in FBS (see Fig. 3B and F depicting SEM images of pellets obtained from FBS and HS, respectively). In addition, the size of the particles seen in the serum pellets was generally smaller than the earlier observed NB produced in DMEM (Fig. 3I–L) which could be due to culture conditions, with the NB having gone through either prolonged cell cultivation in the case of HS or FBS-NB or multiple serial passages in the case of the NB strains “nanons” and DSM 5820 [13].

In the presence of exogenous phosphate, the round particles were seen to rapidly coalesce into aggregates that looked like the granular mattresses seen earlier with dark field microscopy (Fig. 2D and F–H). Different stages of this aggregation could be seen sometimes within the same specimens, with some sections appearing more granular, presumably indicating an earlier stage of aggregation (Fig. 3G and H), while others appearing as filaments or mattresses, into which the individual round particles had presumably fused (Fig. 3C and D).

Control, untreated serum pellets also displayed a whole array of shapes. Most appeared as poorly defined structures with amorphous and heterogeneous shapes (shown for HS-1 in Fig. 3E). However, in all the control pellets that we examined, occasional patches of round particles or mattresses could be seen. Figure 3A shows an example of granular pattern seen with control FBS-1 pellet that is virtually indistinguishable from others that have been treated with calcium or phosphate (compare for instance Fig. 3A with 3B).

These morphologies were remarkably similar to those seen with NB (Fig. 3I–L). For purposes of illustration, we used four samples of NB as control, comprised in turn of two NB cultures derived directly from DMEM inoculated with FBS or HS, and 2 NB strains grown in FBS. The morphology of NB was seen to vary markedly with culture conditions like the length of incubation, the initial inoculum size, and the presence or absence of serum. NB were typically of round shape when first formed and they were retained largely as particles in the presence of 3 to 10% serum (Fig. 3I–L), but they gradually aggregated into needle-containing spindles which in turn gave way to films and mattresses (not shown). In the absence of serum, NB rapidly converted into larger semi-spherical forms that have also been called “shelters,” “dwellings,” and “igloos,” [1], [2], [6], which in turn coalesced further into membranes or films. Some spheroid particles showed a gradual loss of their smooth surfaces, acquiring instead needle projections or whiskers (more noticeable with the two controls HS-NB and FBS-NB seen in Fig. 3I and J). Others, resembling the serum particles shown earlier, displayed the appearance of mattresses in which fusing or fading spherical particles could be barely discerned or were altogether lost, presumably due to a continuous process of aggregation (not shown).

It should be noted that the morphologies described here for the serum pellets and NB are reminiscent of the calcium phosphate nanoparticles seen in other kinds of calcifications, as for example seen with osteoblast cultures [37] or with tumors [38]. Round nanoparticles of HAP remarkably similar to our serum pellets have also been found in association with calcifications found in animals [39].

TEM of unstained serum pellets provided another morphological aspect not apparent from SEM analysis that further illustrated the marked heterogeneity displayed by these serum entities. The round granular shapes of serum pellets (Fig. 4F and G) were seen to aggregate into larger structures (Fig. 4C) that in turn seemed to elongate into ellipsoid shapes or spindles carrying projecting needles or filaments (Fig. 3B, D, and H). Higher magnification showed that the these filamentous structures appeared to be derived from the fusion of the individual spheres following co-linear deposition patterns, while the stacks or spindles of filaments appeared to form from the juxtaposition of the individual needles (not shown). These morphologies closely resembled the so-called “secondary calciprotein particles” (the term “calciprotein particles” is abbreviated as CPP in the present study) formed by fetuin-albumin-mineral complexes that also appear shaped as “prolate ellipsoids,” as described by Heiss and co-workers [30], [40], [41]. Particles resembling secondary CPP and the serum pellets described here have been isolated recently from the ascites of a patient suffering from calcifying peritonitis indicating that such calcium-containing complexes may form in the body under certain pathological conditions when there is excess calcium loading [30].

For the serum pellets, the amount of needle-like crystalline projections increased with the length of incubation after the addition of the various precipitating ions and prior to the centrifugation step used to obtain serum pellets. In fact, serum pellets that had been centrifuged immediately after addition of calcium and phosphate and that had been washed and stored at 4°C for one week also spontaneously converted from round particles to crystalline needle-like patterns, a behavior identical to what we had seen earlier with NB and NB-like particles (data not shown; refer to ref. 13 for a more detailed discussion of this conversion).

As noted earlier, serum that had not been treated with precipitating ions produced very little pellet; this pellet gave principally images of amorphous, poorly-defined shapes with heterogeneous sizes not seen with the other serum pellets prepared by ion loading (see Fig. 3E shown for HS-1 and Fig. 4A for FBS-1). Occasionally, however, in some control pellets, the same ellipsoid spindles carrying stacks of filaments or needles could be seen (Fig. 4E, shown here for HS-1). Thus, although small in size by comparison, the control serum pellets appeared to display the same plethora of shapes seen associated with ion treatment and that include both spherical particle (Fig. 3A) and filamentous spindle (Fig. 4E) morphologies.

The images obtained for these serum pellets were remarkably similar to those collected for bovine and human NB (shown in Fig. 4I–P). In the presence of serum (3–10%), both bovine and human NB appeared as spherical particles (3^rd^ row, Fig. 4I–L, shown here for both HS-NB and FBS-NB as well as for the two strains of NB known as “nanons” and DSM 5820). In the absence of serum or by allowing them to age in culture, the NB particles appeared also to coalesce into spindles and films, (bottom row, Fig. 4M–P), producing images that were similar to those obtained earlier for the serum pellets.

When examined by thin section TEM, serum pellets also displayed either the round particle (Fig. 5E–H) or the spindle-like morphologies (Fig. 5A–D). The progression of morphological change from round particles to stacks of spindle-shaped filamentous networks was a function of the kind of precipitating ions used, with phosphate and a combination of calcium and phosphate giving more readily spindle shapes as compared with calcium treatment alone. In line with earlier results, either longer incubation times following the treatment of serum with precipitating ions or longer storage of the prepared and washed pellets could each enhance the transformation of round particles into spindles and films. In general, however, any of the specimens studied was seen to display a broad spectrum of morphologies. For instance, although the FBS pellets were shown mainly to illustrate filamentous and needle-like crystalline projections (Fig. 5A–D, K and L), in the same specimens round morphologies were also readily seen (Fig. 5I and J). Needle-like projections or whiskers could also be seen radiating from the surface of some particles, which appeared to be undergoing a gradual transformation from round particles to spindles (Fig. 5D and K). FBS pellets obtained through both calcium and phosphate treatments tended to produce stacks or spindles of larger crystalline filaments with longitudinal lengths reaching upwards of 250 nm (Fig. 5D, K, and L). On the other hand, the HS pellets shown here harbored round particles of small sizes with diameters ranging from 20 to 100 nm (Fig. 5E–H). Some of the round particles seen in the HS pellets were found to possess an electron-dense wall with either electron-pervious or dense amorphous core material (Fig. 5G and H) while others appeared to harbor semi-crystalline material with lower electron-density (Fig. 5F and G). With prolonged incubation of serum with calcium and phosphate, the same granulations were seen to also acquire multi-layered walls (not shown). In addition, when serum granulations were transferred to DMEM containing serum (1–10% FBS), the same particles were seen to grow further in size and to acquire eventually concentric layers of alternating densities with prolonged incubation (not shown).

These morphologies were similar to those seen for NB as exemplified here by both NB obtained from serum-containing medium as well as the NB strain “nanons” (Fig. 5M–T). As before, a variety of distinct morphologies could be seen. Small single-walled spherical particles were generally associated with the initial NB population (Fig. 5M). However, with prolonged incubation, there was either a thickening of the spherical walls or a conversion to larger, multi-layered, concentric spheres (Fig. 5N). Alternatively, there was also conversion from spherical shapes to filamentous spindles or stacks (Fig. 5O and P; see also Fig. 5S for an image of “nanons” undergoing transformation from a round particle shape to that of a filamentous spindle). At times, needles or filaments could be seen projecting from the spheres until these became elongated to resemble spindles (Fig. 5O and P).

A laminated and variegated granular morphology, displaying alternating electron-dense and pervious layers resembling “bull's eyes,” could be more clearly seen with NB “strains” that had been maintained through multiple serial passages in the presence of excess serum (5–10% FBS), as illustrated by thin section images of “nanons” shown in the bottom row of Figure 5 (Q–T). In addition to much larger sizes (some reaching micron sizes), “nanons” were seen here to display the characteristic “tree-age-ring-like” laminated morphology typical of NB [2], [28].

Similar images are also associated with calcium granules commonly found in nature [see Ryall's review cited in ref. 29 in which she shows a gallery of thin section images depicting transversal sections of granules obtained from organisms covering a wide range of phylogenetic complexity and that are virtually indistinguishable from the round particles seen here; compare her Figs. 5 and 10 with our Fig. 5]. In contrast however to NB and the many well-established granules found in nature, the serum pellets studied here showed predominantly spherical particle shapes with single walls, with only a minority displaying the variegated, multi-layered laminated morphology. In the case of calcium granules, the alternating electron dense and pervious layers have been interpreted as reflecting the intercalation of organic and mineral layers, respectively [reviewed in ref. 29], a formation that may also require prolonged time of incubation or storage.

It should also be noted that the serum pellets studied here display marked pleomorphism, with morphologies that include granular as well as membranous and filamentous patches, while the calcium granules described earlier were noted mainly for their spherical, laminated shapes [29]. In order to take into account the marked pleomorphism described here, the serum pellets are also referred as “serum granulations” throughout the text so that they may be distinguished morphologically, at least for the time being, from the predominantly spherical laminated shapes associated with the calcium granules described elsewhere [29]. It is likely that in the presence of excess calcium and phosphate the same spherical calcium granules described elsewhere [29] may also continue to sediment apatite and assume pleomorphic shapes that include films and mattresses. In fact, in the case of Randall's plaque found in the kidneys, the granules comprising the plaque not only carry the characteristic laminated and variegated spherical morphologies, but they are also seen as a thin layer or “plaque” that eventually covers the basal membrane of the thin loop of Henlé, spreading beneath the tubular epithelium [29]. More studies will be needed to ascertain whether all round calcium particles will eventually coalesce to form the spindles and films seen here with our serum granulations.

### Chemical composition of serum-derived calcium and apatite granulations

The chemical composition of the serum granulations was established using a combination of spectroscopy techniques. Powder X-ray diffraction (XRD) analysis of the serum granulations yielded a wide repertoire of amorphous (Fig. 6B and C, shown for HS-2 and HS-4, respectively) and crystalline patterns (Fig. 6D and E, shown for FBS-2 and FBS-4, respectively). In general, these patterns reflected a transition from amorphous particles to Ca_10_ HAP crystals. For the small serum pellets obtained from control serum, amorphous patterns predominated (Fig. 6A, FBS-1), but infrequently, HAP crystals were also detected (Fig. 6F, HS-1).

Like SEM and TEM, the XRD analysis also revealed great variability with regard to amorphous and crystalline patterns obtained for the various specimens studied. Thus, in some specimens, amorphous patterns were retained even in the presence of calcium (Fig. 6B, shown here for HS-2) or calcium and phosphate (Fig. 6C, HS-4). Other experiments revealed a gradual transition to HAP, from the acquisition of a monocalcium phosphate complex (Fig. 6D, FBS-2) all the way to the formation of a final Ca_10_ HAP crystal, as indicated by the prominent peak at 31.8 degrees on the 2-θ scale (Fig. 6E, FBS-4). HAP crystals could be detected at times even when the initial serum pellets were obtained from untreated serum samples, as illustrated by Figure 6F, where a small Ca_10_ HAP signal could be seen against the amorphous background. The presence of both amorphous and crystalline forms of calcium phosphate in the same samples confirms the earlier morphological findings of a wide progression of forms spanning from small round particles all the way to films.

These XRD spectra were similar to those seen for NB cultured from 10% HS (Fig. 6G, HS-NB) or from 10% FBS (Fig 6H, shown here for the NB strain “nanons”) which both gave spectra that overlapped entirely with the Ca_10_ HAP peak seen at 31.8 degrees, as also seen with the commercially available powder used as a reference (Fig 6I, HAP). Compared to the HAP control, however, both our serum granulations and NB gave variable XRD spectra yielding either amorphous patterns or a few single peaks, indicating variable, if not lower, degrees of crystallinity (Fig. 6D–F, compare these with the number and intensity of peaks seen for the HAP control shown in Fig. 6I, which revealed a much more complex pattern of peak signals). In the case of the serum granulations, the intensity of these peaks could be modulated by the amount of calcium and phosphate added to the serum as well as by the temperature and length of the subsequent incubation, with greater crystallinity resulting from higher amounts of precipitating ions, longer incubations, and higher temperatures. In the case of NB, the degree of crystallinity varied also with the amount of serum added to the culture medium, with less crystallinity being generally associated with higher amounts of serum (5–10% compared to 1–2%; data not shown). This negative correlation between crystallinity and serum protein content appears to support the notion of a dual inhibitor-seeder role for serum proteins proposed here to explain the formation of both calcium granulations and NB from serum. Finally, it should be noted that the XRD spectra shown here are not unique to serum granulations and NB; similar spectra have been described for extra-skeletal calcification deposits associated with various human diseases [38], [42].

Next, energy-dispersive X-ray spectroscopy (EDX) was done on the same serum pellets obtained from either control serum or from serum treated with calcium, phosphate, or both. As revealed by EDX analysis, untreated serum pellets contained various elements, including calcium and phosphorus (Fig. 7E, compare signals with those obtained for the CaCO_3_, Ca_3_(PO_4_)_2_, and HAP controls shown in Fig. 7L–N) and, at times, unrelated metallic elements (Fig. 7A, with a FBS-1 sample showing the presence of titanium). Upon addition of precipitating ions, the resultant mineral complexes formed by any one of the ion treatments accumulated calcium and phosphorus with Ca/P ratios typically reaching between 1.2 and 1.9 (Fig. 7B–D, F–H), again indicating heterogeneity of calcium phosphate compositions, spanning probably from mono-basic forms all the way to crystalline apatite. Thus, even the treatment of serum with either calcium or phosphate alone resulted in the formation of serum granulations that contained both calcium and phosphorus, attesting to the high binding affinity between calcium and phosphate and consequently their preferential co-precipitation as mineral particles. In the case of serum pellets prepared following serum treatment with calcium alone, the presence of phosphorus gradually increased on EDX spectra as a function of the time of incubation. For instance, a serum pellet obtained after addition of calcium to FBS followed by a short incubation period of 1 hour showed only a small incorporation of phosphorus (Fig. 7O, labeled as FBS-2′; note the low phosphorus peak), but after longer overnight incubation, the amount of phosphorus incorporated into the serum pellet was considerably increased (Fig. 7P, labeled FBS-2″), which now appeared indistinguishable from that seen associated with the other spectra shown earlier. These observations further support the notion that amorphous calcium compounds formed in the absence of phosphorus tend to acquire phosphorus when further exposed to phosphate-containing medium, a process that is expected to continue until HAP is formed, due to the high binding affinities known to exist between calcium and phosphate [13], [16].

Beside calcium and phosphorus, other elements were also detected but these varied with the lots of serum used and did not appear to be representative (Fig. 7A–F, and H). The Ca/P ratios observed for the serum granulations studied here fell within the range described earlier for NB as well as for carbonate HAP associated with bones [2], [13]. Thus, serum granulations were shown to have elemental compositions similar to NB derived from HS (Fig. 7I, with the NB specimen obtained from a culture of DMEM inoculated with 10% HS derived from a healthy donor). Similar EDX profiles were also obtained for NB cultured from 10% FBS, as exemplified by the two representative EDX analyses obtained for the NB strains “nanons” (Fig. 7J) and DSM 5820 (Fig. 7K). All NB samples displayed major peaks of calcium and phosphorus similar to those seen for the serum granulations.

Again, the EDX profiles presented here are not unique to our serum granulations or NB as similar profiles have been associated with calcium granules found in platelets [43] as well as in ectopic pathological calcifications [44]. In all these specimens examined, there was a predominance of calcium and phosphate peaks as revealed by EDX.

The mineral phase associated with NB has been identified earlier as carbonate HAP [1], [2] which is also the mineral found in bones [45], [46]. In our serum granulation samples, a peak at 31.8 degrees on the 2-θ scale, as seen on the XRD spectra and attributed to Ca_10_ HAP, was the main signal detected (Fig. 6E and F). The presence of carbonate in our samples would call mainly for an overlapping signal near 33 degrees on the same 2-θ scale, but this peak is difficult to distinguish from the HAP signal seen near this region [13]. Furthermore, it is not clear whether our samples carry this particular crystalline diffraction plane for carbonate. To verify whether carbonate is indeed present in the serum granulations, Fourier-transformed infrared spectroscopy (FTIR) was used instead. FTIR is known to yield more distinct carbonate and phosphate signals; it also allows for the presence of related amorphous groups to be better distinguished.

Serum granulations obtained from untreated serum or from serum treated with additions of either calcium, phosphate, or both, gave peaks corresponding to carbonate groups as well as phosphate moieties (Fig. 8A–H). Peaks corresponding to carbonate groups could be seen at 875 cm^−1^ and around 1,400–1,430 cm^−1^, with a noticeable split in the asymmetric stretching band seen in this latter position (Fig. 8A–H, compare with the spectrum of CaCO_3_ shown in Fig. 8K; see also refs. 47 and 48) while phosphate absorption peaks were noticed at 575 cm^−1^, 605 cm^−1^, 960 cm^−1^, and 1,000–1,150 cm^−1^ (Fig. 8A–H, compare with signals obtained for Ca_3_(PO_4_)_2_ and HAP shown in Fig. 8L and M, respectively; see also refs. 49 and 50). Although there was some variability in the signal strengths seen with the serum samples, with some producing low or barely detectable signal strengths, there was generally the presence of both carbonate and phosphate peaks in all the serum pellet samples seen.

While all FBS and HS samples, including untreated controls, produced granulations that yielded small or barely detectable signals at 875 cm^−1^, they gave much stronger signals at the 1,400–1,430 cm^−1^ position, indicating the clear presence of amorphous carbonate in these preparations. Compared to the carbonate signals, the phosphate signals were more prominent in all the serum pellets. In the case of untreated FBS granulations, the peak corresponding to carbonate groups at 875 cm^−1^ was absent (Fig. 8A), but the presence of carbonate in this sample was apparent from the other carbonate peak at 1,400 cm^−1^ (Fig. 8A). The intensity of the phosphate absorption bands in untreated FBS pellet was also low (Fig. 8A, compare the intensity of the phosphate bands at 575 cm^−1^, 605 cm^−1^, 960 cm^−1^, and 1,000–1,150 cm^−1^ with those of the other serum pellets in Fig. 8B–H). While these observations would suggest that only a low amount of carbonate and phosphate might be present in the untreated FBS pellet seen here, it should be noted however that there was considerable data variability depending on the serum lot used. The untreated HS-1 pellet for example produced much stronger carbonate and phosphate signals when compared with its untreated FBS-1 counterpart (Fig. 8E, compare with Fig. 8A). The variability seen with the spectroscopy patterns, especially the ones associated with serum granulations derived from untreated serum, suggests in a certain degree of chemical heterogeneity, a scenario which would parallel the marked morphological heterogeneity seen earlier.

Other peaks corresponding to amide I, II, and III, were noticed for the various serum granulations at 1,660 cm^−1^, 1,550 cm^−1^, and 1,250 cm^−1^, respectively (Fig. 8A–H; see refs. 45 and 51). These peaks correspond to the different modes of absorption of the amide bond commonly found in proteins. For instance, the peak of amide I is principally attributed to the stretching vibration of the C = O bond, while the peak of amide II represents the bending of the planar N-H bond as well as the stretching vibration of the C-N bond [51]. Finally, the peak of amide III also corresponds to the bending of the N-H bond [51]. The presence of these different absorption components of the amide bond indicates that proteins are abundantly present in the serum granulations. It should be noted however that the peak corresponding to amide I at 1,660 cm^−1^ could not be distinguished clearly from an overlapping peak at 1,650 cm^−1^ attributed to the presence of the hydroxyl group or residual water [52], [53] (Fig. 8K–M; note the presence of the peak corresponding to the hydroxyl group or residual water at 1,650 cm^−1^ in the controls which would unlikely contain any proteins). The amide III peak on the other hand overlapped with the broad phosphate group seen on its right (Fig. 8E). In contrast, the amide II peak at 1,550 cm^−1^ appeared unique and was clearly seen in all the serum pellet samples (Fig. 8A–H).

The FTIR spectra obtained for serum granulations were next compared to those obtained for NB cultured from 10% HS (Fig. 8I) or for “nanons” cultured in 10% FBS (Fig. 8J). Both serum granulations and NB showed the presence of small carbonate and phosphate peaks at the exact same positions. The peak corresponding to amide I at 1,660 cm^−1^ was also noticed in the NB specimens, but the amide II peak at 1,550 cm^−1^ and the amide III peak at 1,250 cm^−1^ were absent (Fig. 8I and J). These results indicate that the NB specimens studied here may contain significantly less protein than the serum granulation samples, consistent with the fact that these serum granulations had been obtained directly from whole serum while the NB preparations were obtained from DMEM containing only 10% serum, not to mention their prolonged incubation in culture that may have resulted in the progressive degradation of proteins as suggested by our previous study [13]. From this simple comparison, however, it can be inferred that, while serum granulations and NB may differ somewhat in protein content, they appear to have a similar chemical composition insofar as carbonate and phosphate groups are concerned. In this respect, the FTIR data shown here for both serum granulations and NB resemble those collected earlier for calcium granules found *in vivo* within kidney or pancreatic tissues, which also revealed the presence of carbonate and phosphate suggestive of carbonate apatite [54]–[56].

We also verified the chemical composition of the same serum granulations by micro-Raman spectroscopy analysis. Depending on the serum lot used, serum granulations prepared from FBS and HS gave marked variability in the nature of the peaks seen (Fig. 9A–H). For instance, the spectra of the FBS granulations obtained from untreated serum did not show any substantial peak (Fig. 9A) while the serum granulations obtained following the addition of either calcium or phosphate, or both, showed low broad peaks at 962 cm^−1^ characteristic of phosphate ions while the signals for carbonate could not be detected in these same samples (Fig. 9B–D). These results stood in marked contrast to the spectra obtained for control commercial preparations of calcium carbonate as well as the calcium phosphate compounds Ca_3_(PO_4_)_2_ and HAP, as shown in Figure 9K–M [Bibr pone.0005421-deMul1] [see also refs. 57 and 58]. Note that the calcium carbonate control yielded typical signals at 280 cm^−1^, 712 cm^−1^ and 1,080 cm^−1^ (Fig. 9K). As for the phosphate signal, due to the low intensity of the signals observed here, we could not discern the other phosphate ion peaks at 361 cm^−1^, 440 cm^−1^, 581 cm^−1^, 1,048 cm^−1^ and 1,076 cm^−1^ seen in our earlier NB preparations [13] as well as in the Ca_3_(PO_4_)_2_ and HAP controls (Fig. 9L and M, respectively).

The spectra obtained for untreated HS granulations as well as HS granulations obtained following additions of phosphate were similar as judged by the presence of both phosphate and carbonate peaks at 1,002 cm^−1^ and 1,150 cm^−1^, respectively (Fig. 9E and G, with similar signals having been attributed respectively to HPO_4_^2−^ ions in ref. 58 and to CO_3_^2−^ substitutions in the HAP lattice in ref. 59). On the other hand, HS granulations obtained following addition of calcium yielded a low peak of carbonate near 1,080 cm^−1^ whereas the phosphate signals were either absent or barely visible (Fig. 9F; compare with the spectrum of CaCO_3_ shown in Fig. 9K). Finally, following the addition of both calcium and phosphate to HS, the granulations obtained gave low peaks corresponding to phosphate at 440 cm^−1^ and 962 cm^−1^ as well as a low peak of carbonate at 1,080 cm^−1^ (Fig. 9H). It should be noted that some peaks of low intensity seen for example at 1,002 cm^−1^ and 1,150 cm^−1^ in HS serum granulations (Fig. 9E and G) were not observed in commercial control preparations (Fig. 9K–M). Similarly, some peaks present at 290 cm^−1^, 361 cm^−1^, 702 cm^−1^, and 1,048 cm^−1^ in the commercial powders included as references, annotated in Figure 9K–M in accordance with refs. 57, 58, 60 and 61, were rarely seen in our samples. Although phosphate and carbonate peaks were obtained consistently for serum granulations when subjected to the FTIR analysis, it is speculated that the absence of noticeable carbonate and phosphate peaks in some of the Raman spectra may be due to the overwhelming presence of proteins in the serum granulation samples, a notion consistent with published studies [45]. Accordingly, under the influence of the green laser used for the Raman analysis, proteins produce fluorescence which results in the presence of a high background with concomitant dampening of the signal-to-noise ratios related to the specimens [45].

In support of this explanation, the spectra of NB samples cultured from 10% HS (Fig. 9I) or 10% FBS (Fig. 9J) consistently showed peaks of phosphate at 440 cm^−1^, 581 cm^−1^, and 962 cm^−1^ as well as carbonate peaks at 1,080 cm^−1^ that were slightly more noticeable than those produced by serum granulations, perhaps due to a lower protein content associated with NB as compared with serum granulations. In spite of these intensity differences, the positions of the various peaks seen with NB and serum granulation samples appeared virtually indistinguishable. Together with the data obtained through EDX and FTIR analyses, our Raman data further support the notion that the serum granulations and NB have a similar chemical composition.

Similar Raman profiles have also been seen earlier in association with various calcification deposits in humans as in the case of atherosclerosis, which also gave similar signals for carbonate and phosphate groups [62], [63]. These observations suggest that the calcium deposits found *in vivo* share many of the morphological and chemical characteristics of serum granulations and NB described here.

We examined further the crystalline nature of serum granulations by analyzing their electron diffraction patterns obtained by TEM (Fig. 10, insets). Along with the TEM images of serum granulations shown in Fig. 4, the additional images shown in Figure 10 illustrate the marked morphological heterogeneity seen associated with serum granulations (compare Fig. 10 with Fig. 4; note also the typically crystalline morphology of the control commercial preparations of CaCO_3_, Ca_3_(PO_4_)_2_, and HAP now included as controls in Fig. 10L–N, respectively). Both Figures 4 and 10 reveal pleomorphism within the same sample preparations. For instance, the same untreated control FBS-1 sample was shown to harbor both amorphous aggregate shapes (Fig. 4A) as well as round electron-dense particles (Fig. 10A). Likewise, HS-1 obtained from untreated control serum was also seen to contain both granular aggregates (Fig. 10E) and more crystalline spindles (Fig. 4E). Others like HS-2 showed round particles (Fig. 4F) undergoing progressive stages of aggregation and fusion (Fig. 10F).

As can be seen from the diffraction patterns presented in the insets of Figure 10, most serum pellet samples displayed several fuzzy concentric rings of variable intensities characteristic of polycrystalline materials with low degrees of crystallinity (Fig. 10A, B, E–G) while others produced more intense rings with dots indicating a higher degree of crystallinity (Fig. 10C, D, and H). The presence of dots in the diffraction patterns of some serum granulations generally correlated with the appearance of more crystalline particles similar to the secondary CPP seen elsewhere [40], but occasionally even such particles produced patterns with concentric rings without visible dots (Fig. 10B). The fuzzy concentric pattern was independent of the source of serum or the type of serum treatment used. By comparison, the diffraction patterns obtained for the commercial controls consistently showed the presence of discrete arrays of dots (Fig. 10L–N). Such dots indicate a higher intensity of diffraction obtained at specific angles and are usually seen in crystals with a high degree of crystallinity. From this comparison, it appears that the serum granulation samples studied here consisted of polycrystalline materials with relatively low degrees of crystallinity which may be attributed in turn to the presence of amorphous mineral phases undergoing varying degrees of crystallization, as demonstrated by several samples submitted to XRD analysis (Fig. 6A–C). On the other hand, the low crystallinity may also correspond to the presence of carbonate substitutions in the crystal lattice [49]. Diffraction patterns with similar fuzzy concentric rings were also observed in dentin [64] and calcified cartilage [65], as well as in tissues undergoing extraskeletal calcification [66], which were also interpreted as the presence of HAP possessing a low degree of crystallinity. As a further reference, diffraction patterns of the mineral precursor in bones produced amorphous diffraction patterns while more mature bone harbored both fuzzy rings and dots similar to the more crystalline patterns obtained for some of our serum granulations [67]. This suggests that calcium granulations found in the serum resemble closely calcium phosphate deposits found in the human body in both normal and pathological conditions.

### Identification of proteins associated with serum-derived calcium and apatite granulations

**Figure 11:**
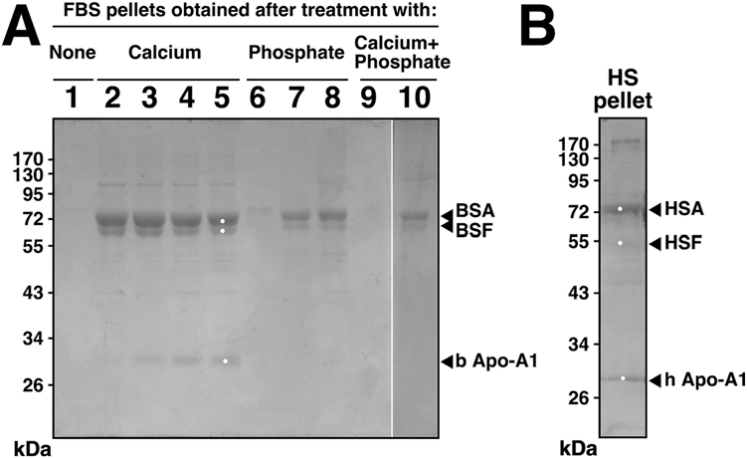
SDS-PAGE profiles of proteins bound to serum granulations and protein identification. (A) Serum granulations (pellets) were prepared exactly as in Fig. 1, from either untreated FBS (referred as “None”) or through the addition of the indicated amounts of CaCl_2_ (“Calcium”) and/or Na_2_HPO_4_ (“Phosphate”) to FBS. The indicated sequence of the lanes depicted in the heading matches exactly with the sequence of wells seen in Fig. 1. No protein band was found in the pellet of untreated FBS (lane 1), whereas the other lanes showed dose-dependence with respect to the precipitating reagents used. The white marks represent the positions of the bands excised for identification by MALDI-TOF mass fingerprint analysis whereas the identified proteins are shown on the right. While bovine serum albumin (BSA) and apolipoprotein A1 (b Apo-A1) were the only proteins identified in their respective positions, other proteins besides bovine fetuin-A (BSF) were also identified in the 52–65 kDa range (see the text for details). (B) This HS pellet was obtained with 30 mM CaCl_2_ and processed exactly as done with the FBS pellets. Abbreviations used: HSA, human serum albumin; HSF, human serum fetuin-A; and h Apo-A1, human apolipoprotein A1.

By SDS-PAGE analysis, the protein profiles associated with serum granulations consisted of three main bands (Fig. 11). Lanes 1 through 10 of Figure 11A, shown for FBS granulations, represent the exact SDS-PAGE profile counterpart of Figure 1. No protein band was seen in the untreated FBS pellet (Fig. 11A, lane 1). Increasing the amount of calcium (lanes 2–5) or phosphate (lanes 6–8) or calcium and phosphate (lanes 9 and 10) produced a dose-dependent increase in the intensities of protein bands associated with the granulations. Three major bands of 66–75 kDa, 52–65 kDa, and 27–33 kDa were seen with the granulations obtained from both FBS (Fig. 11A) and HS (Fig. 11B), similar to what had been seen earlier with serum NB [13], [15]. The three major bovine proteins were identified by MALDI-TOF mass spectrometry as albumin (3 out of 3 trials), fetuin-A (1/10), and apolipoprotein A1 (2/3), matching exactly with the NB proteins identified earlier through SDS-PAGE [13].

As shown before for FBS-NB [13], and in contrast to another published study [15], fetuin-A did not represent a major protein species even though fetuin-A is one of the more abundant proteins in FBS and represents one of the most avid binders of apatite [see refs. 35 and 68 for excellent reviews on fetuin]-[A]. Instead, several other proteins were also identified in the same fetuin-A position: coagulation factor X (3/10), cytoplasmic actin 1 (2/10), cytoplasmic actin 2 (1/10), myotubularin-related protein 9 (1/10), adenosylhomocysteinase (1/10), and tubulin β-2C chain (1/10). Multiple proteins were also found in the other band positions. Thus, for the 27–33 kDa band, flotillin-1 (1/3) was also identified in addition to bovine apolipoprotein A1.

For the HS pellet, albumin (2/2), fetuin-A (1/1), and apolipoprotein A1 (1/1) were also identified as indicated in Figure 11B, with the three proteins labeled as HSA, HSF, and h Apo-A1, respectively. These three proteins were also seen as the main protein bands associated with HS-NB [13]. From the gel profile displayed in Figure 11B, it can be seen that while albumin and apolipoprotein A1 represent indeed the major protein bands visible by Coomassie blue staining, the fetuin-A (HSF) band was much less visible. This observation is again consistent with the findings made earlier with HS-NB [13].

In that earlier study [13], we found that several large molecular weight proteins were not dissolved by our gel sample buffer containing EDTA, SDS, and reducing agents and thus these same proteins were found not to migrate into the resolving protein gel. To circumvent this issue, we used a two-step procedure of in-solution-trypsin digestion followed by reversed-phase high performance liquid chromatography (HPLC) to identify the serum pellet-bound proteins, obviating the need to subject the mineral-bound proteins first to SDS-PAGE [13]. Using this approach, we were able to obtain a more extensive determination of the proteins associated with serum granulations. This approach also allowed us to identify several additional high molecular weight proteins that could not be seen on the gel electrophoresis presented in Figure 11.

The main proteins obtained using this alternative proteomic approach are displayed in Tables 1 and 2. Table 1 shows the protein data for FBS granulations and FBS-derived NB while Table 2 displays the proteins obtained for HS granulations and HS-derived NB. In order to have a qualitative indication of the relative abundance of each protein within a sample [69], the amount of unique peptides obtained for each protein was first divided by the total amount of unique peptides obtained for the first 50 proteins in the sample; the number 50 was decided arbitrarily for purposes of standardized normalization and comparison. The value obtained was then multiplied by 100 to obtain a relative value reflecting the abundance of each protein. The order of the proteins presented was based on the sum of the values obtained for each protein identified for all four FBS granulations as well as the four types of FBS-NB used, and the total value derived was in turn used to produce the ranking sequence seen in Table 1 (shown here for only 12 proteins). In the case of human samples, this same sequence was based on the summation of frequency values obtained for the 4 HS granulations and the HS-NB sample used (Table 2). The order of proteins shown in both Tables 1 and 2 stood in general agreement with the relative abundance of each protein studied within any given sample, but there were exceptions to this rule. For example, in Table 1, prothrombin in the FBS-4 sample was more abundant than complement component 3, whereas in the other three FBS granulations, this order was reversed. It is also possible that using this methodology some large proteins may have scored higher than other smaller proteins due to their containing likely more trypsin cleavage sites and consequently generating more associated peptides that may have skewed the ranking frequency in their favor. Nonetheless, the relative abundance derived here provides a useful reference regarding the relative distribution of the proteins found in the various serum granulation samples.

**Table 1:** Proteins of FBS serum granulations and nanobacteria identified by MALDI-TOF mass spectrometry.

#	Identified Proteins^a^	MW^b^ (kDa)	Serum Granulations^c^				Nanobacteria^d^			
			FBS-1	FBS-2	FBS-3	FBS-4	FBS NB	Nanons	DSM 5820	DSM 5821
1	Serum albumin	69	15	11	12	10	27	11	9	17
2	Complement component 3	187	11	6	10	5	13	6	6	5
3	Prothrombin	71	3	5	5	9	2	8	8	6
4	Fetuin-A	38	5	4	4	4	5	5	6	6
5	Apolipoprotein A1	30	5	4	3	3	8	3	4	6
6	Coagulation factor V	249	4	5	7	3	0	5	4	3
7	Hemoglobin fetal subunit β	16	6	3	4	3	5	4	6	5
8	Vitamin K-dependent protein S	75	4	4	1	7	0	6	7	1
9	Thrombospondin-1	130	6	3	5	4	0	6	0	0
10	α-1-antiproteinase	46	6	4	4	2	5	4	0	2
11	Hemoglobin subunit α	15	2	2	1	1	3	2	2	1
12	Adiponectin	26	0	2	1	1	0	1	0	0

a The values shown for each protein correspond to the number of unique peptides identified for each protein which was divided by the total number of peptides obtained for the first 50 proteins obtained in one sample. This value was then multiplied by 100 to reflect the relative abundance of each protein (see the text for more details).

b MW: molecular weight.

c Serum granulations (pellets) were obtained from untreated FBS (FBS-1) or following addition of either CaCl_2_ (FBS-2), Na_2_HPO_4_ (FBS-3), or both (FBS-4) as described in the *Materials and Methods*.

d The different NB samples presented here were all obtained from FBS as described in the *Materials and Methods*.

**Table 2:** Proteins of HS serum granulations and nanobacteria identified by MALDI-TOF mass spectrometry.

#	Identified Proteins^a^	MW^b^ (kDa)	Serum Granulations^c^				Nanobacteria^d^
			HS-1	HS-2	HS-3	HS-4	HS NB
1	Complement component 3	187	8	10	8	15	12
2	Serum albumin	69	11	8	5	6	14
3	Apolipoprotein B100	516	8	6	11	8	6
4	Complement component 4A	193	7	4	4	7	6
5	Apolipoprotein A1	31	2	8	6	5	4
6	α_2_-macroglobulin	163	3	4	5	1	10
7	IGHM protein	53	3	2	2	1	2
8	Serotransferrin	77	3	0	0	0	4
9	Apolipoprotein A2	11	1	2	1	1	1
10	Haptoglobin	47	1	2	2	0	0
11	Fetuin-A	39	0	1	1	1	1
12	Hemoglobin subunit β	16	1	0	0	0	0

a The numbers shown for each protein correspond to the amount of distinct peptides obtained for each protein which was divided by the total amount of distinct peptides identified in the first 50 proteins found in each sample. This value was then multiplied by 100 to give an indication of the relative abundance of each protein.

b MW: molecular weight.

c Serum granulations (pellets) were obtained from untreated HS (HS-1) or following addition of either CaCl_2_ (HS-2), Na_2_HPO_4_ (HS-3), or both (HS-4) as described in the *Materials and Methods*.

d HS NB was obtained from human serum as described in the *Materials and Methods*.

As seen in Table 1, the protein composition of the different FBS granulations was similar, with albumin (ranked the most abundant protein), fetuin-A (ranked 4^th^), and apolipoprotein A1 (5^th^) representing among the five main proteins identified in each case. The presence and relative abundance of these three proteins are in agreement with the protein gel profiles presented earlier (Fig. 11). However, in addition to these three proteins, complement component 3 (ranked 2^nd^) and prothrombin (3^rd^), were also identified that were not obvious from the SDS-PAGE analysis. In other words, with its relative abundance, complement component 3 should have appeared as a noticeable band of 187 kDa in the SDS-polyacrylamide gel while prothrombin (71 kDa) should have been detected in the albumin region after gel excision and protein determination, but as seen from Figure 11, this was not the case however. From Table 1, several other serum proteins (molecular weights ranging between 15 kDa to 249 kDa) were likewise identified but had not been detected by SDS-PAGE. At this time, it is not clear whether the discrepancy seen between the two protein identification methodologies can be explained by the possibility that some proteins may have remained strongly bound to the apatite particles, precluding them from being released into the gel sample buffer in spite of harsh treatment with excess ionic detergents and EDTA as well as boiling. From the data shown here, however, it can be seen that calcium granulations obtained from control, untreated serum (FBS-1) are virtually identical to those collected following treatment of serum with excess calcium (FBS-2) or phosphate (FBS-3), or a combination of both (FBS-4).

Albumin, complement component 3, prothrombin, fetuin-A, and apolipoprotein A1 also appeared among the main proteins obtained for FBS-NB (Table 1). The protein composition of NB obtained from the slow culturing of DMEM inoculated with 10% FBS was remarkably similar to that of the three strains of NB used). These in turn were virtually indistinguishable from granulations obtained from either untreated serum or serum treated with excess calcium, phosphate, or both, indicating similar biochemical compositions for all of them (compare the various protein abundance values seen in Table 1). This finding is significant since the NB specimens studied here appeared to have less protein content when compared with our serum granulations, as suggested by the FTIR analysis shown in Figure 8. That serum granulations and NB should have similar, if not identical, protein compositions as revealed by two different proteomic techniques used here would indicate more of a quantitative rather than qualitative difference associated with the various calcium granulation and NB specimens.

For HS granulations, the three proteins albumin, complement component 3 and apolipoprotein A1 were also among the more abundant proteins identified (Table 2). However, apolipoprotein B100 and complement component 4A were also identified as two of the more commonly associated proteins (Table 2). In contrast, fetuin-A and prothrombin were found at much lower frequencies in the HS granulation samples as compared with the FBS granulations (prothrombin ranked 27^th^ among proteins found in HS granulations and therefore was not listed in Table 2). As seen with FBS granulations, the serum granulations obtained from control, untreated HS displayed a protein composition that was virtually identical to that of the various granulations obtained from HS after loading with calcium (HS-2), phosphate (HS-3), or both (HS-4), indicating again that chemically identical granulations are found in normal serum, albeit at much lower amounts, and that the formation of these entities becomes magnified when the serum becomes loaded with calcium and/or phosphate.

The various HS granulation samples were next compared to the NB cultured from DMEM inoculated with 10% HS (Table 2). With the exception of α_2_-macroglobulin, which turned out to be among the three most abundant proteins for human NB, all other proteins appeared to follow the same frequency pattern shown for HS granulations (Table 2), indicating once again that the HS granulations and NB seen here are probably identical entities.

In addition to the high molecular weight proteins identified and shown in Tables 1 and 2, several others were identified among the 50 most abundant proteins: apolipoprotein(a) (501 kDa; HS granulations), fibronectin (257 kDa; FBS granulations), coagulation factor V (249 kDa; FBS granulations). Several other smaller calcium-binding proteins were also found to be associated with the mineral phase, including vitronectin (54 kDa; HS granulations), vitamin-D-binding protein (53 kDa; both FBS and HS granulations), and kininogen-1 (48 kDa; HS granulations). These proteins were identical to the ones found associated with NB obtained from cell cultures [13]. The results presented here overlap somewhat with earlier studies done to identify the proteins bound to the bone mineral. In one particular study using a similar proteomic approach, the most abundant proteins of bovine bone extracts found to bind the mineral phase of a HAP column also turned out to be albumin and fetuin-A [70]. In fact, albumin has been found at a concentration of 0.7 mg/g of bone in bovines [71] whereas fetuin-A has been found at a concentration of 0.9 to 1 mg/g of bone in rats [72], bovines [71], and humans [73]. In addition, several proteins present in the bones such as decorin, biglycan, osteoadherin, and osteonectin were also identified by the use of a proteomic approach similar to what we have done here [70].

The protein profile obtained for the *Nanobacterium* sp. strain “nanons” deserves some mention. As can be seen from Table 1, “nanons” contained a number of proteins and not just the two proteins fetuin-A and apolipoprotein A1 described by Raoult et al. [15]. This difference in results could very well be accounted by the different techniques used. Like Raoult et al. [15], we have also found that a limited number of bands (in our case, three main protein bands) is seen by SDS-PAGE while multiple proteins can be identified using the direct trypsin-digestion method outlined here. Using both this alternative methodology as well as SDS-PAGE, we have found however that the protein albumin was more commonly observed than fetuin-A. This conclusion is apparent from the data collected from all our serum granulation samples as well as from the other NB specimens studied, including the two strains DSM 5820 and 5821 (Table 1). This same conclusion is further supported by the finding that fetuin-A represents one of the less abundant proteins found associated with human NB and HS granulations (Table 2). In retrospect, this is not surprising given the fact that fetuin-A is found in the adult human serum at levels that are at least 14–26 times lower than those found in FBS (0.7–0.8 mg/ml in HS vs. 10–21 mg/ml in FBS, according to refs. 74 and 75). On the other hand, albumin is known to be present at concentrations as high as 35–45 mg/ml in HS [76] and 23 mg/ml in FBS [77], explaining its predominance in the composition of the serum granulations obtained. Thus, the relative distribution of proteins in the serum granulation scaffold appears entirely circumstantial, a notion that we have proposed also for NB [13].

The presence of various apolipoproteins associated with serum granulations is also noteworthy. Apolipoprotein A1, a protein constituent of high-density lipoproteins (HDL) [78], was abundantly found in the granulations obtained from both adult human serum and FBS. On the other hand, apolipoprotein B100, a well-known constituent of low-density lipoproteins or LDL [78], was among the more common proteins found attached to the human serum granulations. Other apolipoproteins obtained include apolipoprotein A2 (11 kDa, HS granulations) and apolipoprotein(a) (501 kDa, HS granulations) which are found in HDL and lipoprotein Lp(a) particles, respectively [78]. At this time, it is not clear whether the association of serum granulations with the various apolipoproteins is also circumstantial, reflecting merely their relative abundance in the serum, or whether there is a selective enrichment for the apolipoprotein family of proteins in both serum granulations and NB [see also ref. 13]. Given the observations that serum lipids bind to these same particles and that several phospholipids have been shown to nucleate apatite formation [14], [79], it is also possible that circulating lipoproteins in our serum samples may very well anchor apatite, perhaps accounting for at least some of the apatite-seeding activity seen here. In support of this possibility, granulations obtained from untreated serum (both HS and FBS) were seen to be quite heterogeneous, as exemplified through the various ultramicroscopic images shown. More studies will be needed to assess the role of lipoprotein particles in the formation of the serum granulations described here.

### Functional involvement of proteins in both inhibition and seeding of serum granulations

**Figure 12:**
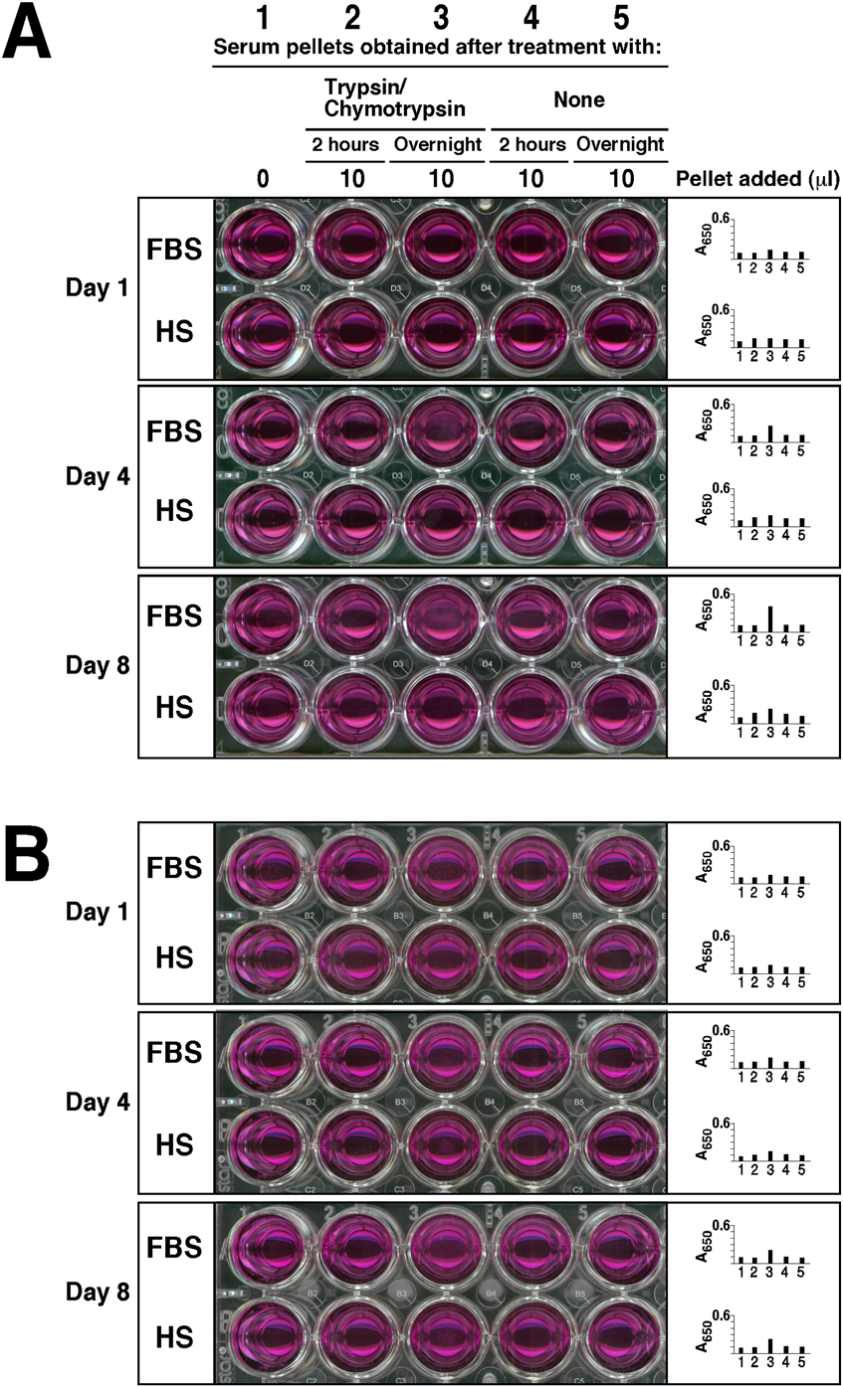
Trypsin and chymotrypsin treatment of FBS and HS generates pellets that have particle-seeding activity when inoculated into serum-free medium. Seeding-pellets were prepared by adding trypsin (A) or chymotrypsin (B) at 0.5% to either FBS or HS, followed by incubation at 37°C for 2 hours or overnight, then centrifugation and washing as described in the *Materials and Methods*. 10 µl aliquots of these resuspended pellets (wells 2 and 3, with well 2 corresponding to 2 hours of serum incubation after protease treatment and well 3 corresponding to overnight incubation of the same protease treatment) were then inoculated into 1 ml of DMEM and incubated in cell culture conditions for the time indicated on the left. “Day 1” refers to the day of pellet inoculation. As control, sera that received no addition of proteases were also incubated, centrifuged, and washed the same way, after which the pellets (barely visible in most control specimens) were inoculated into DMEM and incubated exactly as before (wells 4 and 5, described as “None”). As an additional control, DMEM alone was incubated without any inoculation (well 1). Note the time-dependent increase in turbidity associated with either protease treatment, more noticeable with the pellets that had been obtained from sera treated with proteases overnight as contrasted with 2 hour treatment (compare between visuals obtained for wells 3 and 2). Turbidity was usually higher with the FBS pellets compared to HS pellets. Note also the different amounts of turbidity seen with trypsin (A) vs. chymotrypsin (B) treatments; turbidities were generally higher with trypsin treatment. Control wells inoculated with pellets obtained from serum that received no protease treatment (wells 4 and 5), or containing DMEM alone (well 1), produced either little or no visible turbidity.

The identification of a number of discrete proteins associated with both FBS and HS granulations prompted the question of whether similar circulating serum proteins may play a functional role in keeping calcium and phosphate soluble in the serum under normal, untreated conditions. To address this question, both FBS and HS samples were treated with 0.5% trypsin, after which the trypsin-treated serum was incubated for either 2 hours or overnight at 37°C, followed by centrifugation exactly as before at 16,000×*g*. After 2 hours of incubation, little or no pellet could be discerned, but with longer overnight incubations, small pellets appeared with both trypsin-treated FBS and HS. Upon inoculation of these pellets into serum-free DMEM, it could be seen that the pellets obtained from an overnight trypsin-treatment produced particle seeding following incubation, as evidenced by the clouding of the medium seen both visually and by A_650_ reading (Fig. 12A, “Day 4” panel, wells 3). This seeding effect was more noticeable with the FBS pellet compared to the HS pellet (Fig. 12A, “Day 4” panel, wells 3; compare the amount of pellet and A_650_ reading for these 2 samples). The same particle-seeding effect was time-dependent and increased over a course of several days, ruling out the possibility that the initial pellet could have accounted for the turbidity readings (compare the various panels examined on “Day 4” and “Day 8” with the panel labeled as “Day 1” referring to the first day of pellet collection and seeding). In contrast, neither control DMEM (Fig. 12A, wells 1) nor untreated control serum (Fig. 12A, wells 4 and 5) produced any significant increase in turbidity under these conditions. When analyzed by SDS-PAGE, the trypsin-treated pellets did not produce any visible protein band, but, morphologically, the granulations produced under these conditions were virtually indistinguishable from those seen without trypsin treatment (data not shown). Thus, a variety of shapes that included round particles, spindles, and even films, were seen associated with these pellets (not shown).

A similar precipitation and particle-seeding effect was observed with chymotrypsin (Fig. 12B; compare the amount of pellet and A_650_ readings of the “Day 4” panel, wells 3, with the equivalent wells seen in the “Day 1” panel, obtained on the day of pellet inoculation). In our hands and compared to trypsin, chymotrypsin was less effective in producing serum pellets, with the incubation done under identical conditions. As expected, the amount of subsequent particle seeding produced by serum granulations obtained after chymotrypsin treatment was also significantly lower (compare the wells in column 3 of the “Day 4” and “Day 8” panels in Fig. 12B with their equivalents in Fig. 12A). However, as in the case of the trypsin experiment, the turbidity seen with the chymotrypsin treatment also increased progressively with the length of incubation, indicating an active seeding effect that resulted probably from cumulative nucleation and growth of HAP (Fig. 12B).

These results indicate that protease-sensitive protein moieties appear to contribute to the maintenance of calcium and phosphate solubility in the serum. That is, protease treatment of both FBS and HS alone resulted in precipitation, a result that became more pronounced when the serum incubation with the protease was lengthened from 2 hours to overnight. The pellets obtained under these conditions were then further shown to have particle-seeding or proliferation activity upon subsequent inoculation into fresh medium. That the protease-treated serum should generate insoluble precipitates is in line with earlier reports addressing the role of major serum proteins like albumin [80] and other apatite-binding proteins [81] in keeping both calcium and apatite in solution in the serum. The protease experiments also demonstrate that the serum precipitates obtained in the absence of these proteins appear to resemble morphologically the serum granulations formed in the presence of intact serum proteins (comparative data not shown), suggesting that perhaps there are other organic factors (and even peptide fragments) remaining in the serum that may similarly modulate and create the same pleomorphic granulation shapes.

It should also be noted that in a previous study [13], we had shown that trypsin treatment of serum enhanced the production of NB-like particles when the same serum was inoculated into fresh medium. Based on our findings here, however, it can be inferred that the treatment of serum with proteases results in the direct precipitation of apatite complexes which in turn exert a particle-seeding effect when transferred into fresh serum-free medium. While our results do not address the role of any specific protein(s) in this process of precipitation, our findings do point to a direct chemical binding phenomenon involving both proteins and minerals that may form the basis for the dual inhibition and seeding mechanisms deemed necessary for the assembly of the calcium/apatite granulations studied here. Similar mechanisms may also be involved in the assembly of entities that most likely have become misconstrued as living microorganisms or as calcifying agents of disease known as NB. More importantly, our results also suggest that these same mechanisms may be widely used in nature for the generation of similar calcium and apatite granules.

### Conclusion and future perspectives

The results shown here demonstrate that the protein scaffold and its main constituents found associated with NB [13] can now be reproduced directly with whole serum. That is, we are able to reproduce here the NB phenomenology in its entirety using only serum and without the need for the prolonged incubations in cell culture medium previously deemed necessary for the growth and demonstration of NB. The proteins identified here as part of serum granulations also represent the more abundant serum proteins that happen to bind avidly to both calcium and apatite [13]. This protein binding to calcium and apatite may represent a general mechanism of protection deployed within the blood compartment and possibly within all the other body fluids that serves to cope with calcium and/or phosphate loading. In this sense, the calcium granulations seen here can be viewed as by-products or remnants of calcium and apatite binding reactions that may likely become amplified through calcium and phosphate loading. Along this vein of reasoning, perhaps the same pathways may also be amplified artificially in culture mediums exposed to serum proteins and saturating levels of calcium and phosphate, which may have given the erroneous past impression of supporting the existence of exotic organisms or perhaps novel pathogenic entities referred as NB [1]–[8].

Serum has long been known to contain potent inhibitors of spontaneous calcium and apatite precipitation [82]. Serum proteins mediating apatite inhibition include both calcium-binding proteins like albumin and apatite-binding proteins like fetuin-A [81]. In this respect, our previous study had shown a paradoxical inhibitory effect of serum on NB formation that also turned out to be trypsin-sensitive [13]. Preliminary results using the NB-like precipitation system developed in that study [13] suggest that the addition of excess calcium and/or phosphate to serum, even at the high concentrations used, may not have exhausted the full inhibitory (and seeding) capacity of the serum studied and that only a fraction of the total inhibitory components has been consumed during this process (not shown). The possibility also exists that other calcium- and phosphate-binding factors, including non-proteinaceous components, may be present in the serum in large amounts and that these factors may remain soluble even in the presence of excess calcium or phosphate. In fact, small inorganic compounds like pyrophosphate have been shown to inhibit effectively apatite formation [83], [84]. The chemical structure of pyrophosphate has led to the development of synthetic calcification inhibitors like bisphosphonates [83], [85], now used as treatments for the prevention of mineral loss in osteoporosis [86].

It appears that the calcification inhibitory mechanisms seen in the serum, as evidenced from our study here, are geared to effectively bind both calcium and phosphate. When both calcium and phosphate are present in the serum, the inhibitory complexes appear to reach saturation with ion concentrations ranging between 1 to 3 mM each, above which there is the formation of precipitating complexes and granulations resembling the putative NB. The range of ion concentrations that induces a transition from solubility to precipitation may vary with the particular conditions used, as exemplified by experiments using longer periods of incubation from 1-to-7 days, whereby even calcium phosphate concentrations under 1 mM could be shown to form NB-like complexes (not shown). On the other hand, when either calcium or phosphate is added individually to both FBS and HS, saturation is not reached until much higher amounts of either ion species are present, indicating that the inhibitory mechanisms inherent in the serum can be effectively overcome only when both calcium and phosphate are simultaneously present. Short of their dual presence, it is more likely that a state of calcification-inhibition would prevail instead.

The need for both calcium and phosphate to be present to disarm this same calcification-inhibition reinforces the known concept of ion-product (that is, calcium ion concentration×phosphate ion concentration) used to determine the propensity for calcification in any given fluid system [83]. Any solution at physiological pH, ionic strength and body temperature, which would include serum in body conditions, has been found to spontaneously precipitate when its ion-product becomes equal to or greater than 6×10^−6^ M^2^ [83]. This number is far above (in fact, by at least 22 orders of magnitude) the chemical equilibrium calculated from the solubility product of synthetic HAP as related to its calcium and phosphate constituents. That is, calcium phosphate has far greater propensity to nucleate HAP than what is actually seen in biological systems like serum. The presence of serum inhibitors clearly removes this thermodynamically-favored propensity toward calcification! Thus, with known HS concentrations for calcium and phosphate averaging 1.2 mM and 1.3 mM, respectively [83], the normal ion-product is 1.56×10^−6^ M^2^, still below the ion-product threshold for calcification. However, the ion-product concept cannot fully explain the different susceptibilities of serum to precipitate in response to the various precipitating ions added. Thus, our experiments demonstrate that while the precipitation-threshold level of 6×10^−6^ M^2^ for the ion-product can be exceeded by adding either calcium, phosphate, or a combination of both, to give the same final ion-product, the simultaneous addition of both calcium and phosphate is much more effective in inducing HAP formation than the addition of either calcium or phosphate alone. Accordingly, addition of calcium alone to HS giving an ion-product that exceeds 6×10^−6^ M^2^ by as much as 30% has shown to produce little or no precipitation. Along the same line of reasoning, phosphate is more effective than calcium, when added to the same final ion-product. Furthermore, precipitation occurs with the addition of only 1 mM of both calcium and phosphate to serum that would give in turn an ion-product of 5.06×10^−6^ M^2^, still below the threshold level for precipitation, provided only that a prolonged incubation is used. It can be inferred therefore that the calcification-inhibitory processes inherent in the serum are geared to dealing more efficiently with loadings of either calcium or phosphate alone and are overcome much more easily with the simultaneous presence of excess calcium and phosphate.

Given that the body fluids are supersaturated with respect to calcium and phosphate ions [30]–[35], it can be surmised that the same inhibitory factors and mechanisms must be at work to maintain the solubility of these ions in the body fluids. It follows that upsetting this balance, which would happen when the same inhibitory state is finally overcome, should result in a likewise precipitation of calcium phosphate and the formation of HAP, which would then have to be disposed from the body in order for calcification to be avoided. Since the blood levels of calcium and phosphate are known to fluctuate even under normal conditions, presumably the formation of a small amount of calcium phosphate complexes can be expected to occur in the healthy state. These ion fluctuations are probably more manifest locally in certain tissues like the kidneys or the bones which experience a constant remodeling process [83]. In a healthy state, these nascent mineral complexes are likely to get removed from the serum by binding to calcification inhibitors like fetuin-A and albumin, followed by phagocytosis by the reticuloendothelial system (RES), or perhaps via excretion in the urine [30], [40], [83]. Assuming that the inhibitory system is overwhelmed as, say, in the case of cardiovascular or late-stage kidney diseases, a more extensive deposition of calcium phosphate complexes in the body may in principle contribute to the disease process, and anomalous calcification has indeed been seen in the disorders cited as examples here [87]–[89]. However, it appears that the process of granule formation described here can be explained by the workings of normal homeostatic mechanisms that regulate calcium and apatite in the body and that seek to prevent anomalous calcification.

In this context, the large number of proteins bound to the calcium granulations obtained from both HS and FBS, along with the earlier spectroscopy data suggesting an abundant presence of bound proteins, indicate that the apatite formation seen here corresponds probably to a process of “heterogeneous nucleation”—the formation of apatite crystals on a protein template [83]. While this term has been used largely in the past to describe calcification seen associated with collagen fibrils and other tissue- bound or immobilized proteins as contrasted with spontaneous, “homogenous nucleation” seen with calcium phosphate exceeding the precipitation-threshold levels for the ion-product [83], it is likely that the apatite nucleation seen here involves a similar process of heterogeneous nucleation since the same protein inhibitors that bind to calcium or nascent calcium phosphate crystals may in fact turn out to be the very anchors or seeds used for further apatite growth once they undergo saturation and conformational change. Finally, the resultant protein-mineral phase transformation is likely to be temperature and time-dependent, which would be consistent with the observations made here and elsewhere [13]. That is, following binding to calcium or apatite and after undergoing the necessary conformational change, the soluble calcification-inhibitor presumably becomes insoluble, turning into a bona fide template for further apatite nucleation. That any calcium and apatite-binding protein can act in principle as both inhibitor and nucleator of apatite formation is actually supported by extensive studies done on a number of proteins with recognized roles in biomineralization [see the excellent review by Benesch et al., ref. 90]. Thus, in addition to fetuin-A and albumin, proteins like amelogenin, decorin, bone acidic glycoprotein 75, bone sialoprotein, chondrocalcin, fibrinogen, fibronectin, matrix G1a protein, osteocalcin, osteonectin, osteopontin, statherin, vitronectin, and various other phosphoproteins have all been shown to display apatite inhibitory and/or nucleating activities, with the predominance of one or the other activity depending on both the *in vitro* or *in vivo* conditions used, including whether the proteins were tested soluble or immobilized on solid substrates [90].

The presence in the serum granulation and NB scaffold of proteins like albumin, complement components 3 and 4A, prothrombin, fetuin-A, apolipoproteins A1 and B100, as well as other calcium and apatite binding proteins, appears to be entirely circumstantial, depending on their availability in the serum milieu. This conclusion is supported by the presence of select proteins in the granule scaffold that depend solely on the particular species and type of serum used (prothrombin in FBS granulations versus apolipoprotein B100 in HS granulations; more of fetuin-A in FBS granulations compared to HS granulations). That is, the protein profile seen here reflects largely the relative abundance of these same proteins in the serum used. Thus, albumin, the predominant protein identified here, is not only one of the more abundant serum proteins but is also known to account for about half of the calcium-binding activity measured in HS [80]. Likewise, fetuin-A, an avid apatite-binding protein, accounts for about half to one-third of the total serum inhibition of the spontaneous precipitation of apatite seen with supersaturated solutions of calcium and phosphate [91]. Extending the model presented here, the protein profiles of calcium granulations formed in the various body fluids and tissues are predicted to largely reflect the composition of the major calcium and apatite binding proteins available in the surrounding milieu, a notion that had been confirmed through *in vitro* experiments done on several body fluids challenged with calcium phosphate loading [13].

The formation of mineral complexes in pathophysiological conditions has been illustrated recently by the description of calciprotein particles, and more specifically, by the so-called “secondary CPP,” which have been isolated from the ascites of a patient suffering from calcifying peritonitis linked to kidney disorders [30]. CPP are colloidal calcium phosphate particles shown to transform from small, soluble, round particles (usually smaller than 150 nm) to larger, less soluble, and more crystalline “prolate ellipsoids” [30], [40], [41], [83]. Crystalline needle-like projections appeared on the round amorphous CPP within 6 hours of incubation at 37°C, but a longer period of incubation of at least 30 hours was usually needed for a complete conversion of round CPP into more crystalline secondary CPP [40], [83]. The formation of CPP *in vitro* requires the presence of fetuin-A for their formation and stabilization, but the CPP found *in vivo* contain a low amount of fetuin-A [30]. Instead, it appears that fetuin-A can be substituted, at least in part, by other acidic serum proteins like albumin, and in fact, albumin is present in high amounts in the CPP isolated *in vivo* [30]. Fetuin-A present on the surface of the CPP appears to prevent further growth and aggregation of the particles, perhaps by reducing the diffusion of mineral ions inside the particles [41], [92]. According to Heiss, Jahnen-Dechent, Schäfer, Ketteler, and their colleagues, fetuin-A, and perhaps other acidic proteins, should be viewed as “mineral chaperones,” being able to lead to the transient formation of a soluble mineral complex for the transport and clearance of calcium and phosphate ions under conditions of local supersaturation, a role that these authors deem analogous to the blood transport of lipids inside lipoprotein particles [30], [68]. With time, however, this same soluble complex undergoes phase transformation becoming insoluble and precipitating out as crystalline apatite nuclei [30], [40], [41]. Based on our own study, this same phase and solubility transformation may also explain the dual inhibition-nucleation concept proposed here as the basis for the formation of serum granulations resulting from calcium phosphate loading.

Evidence that mineral complexes similar to the ones described in this study may form in pathological conditions also comes from the observation that treating rats with etidronate, a biophosphonate known to decrease the mineralization of bones, or vitamin D, a liposoluble vitamin known to be required for the absorption of intestinal calcium and phosphate ions, led to the formation of protein-mineral particles in the blood of rats [93]–[96]. Treatments with both molecules led to increases in the total serum concentration of calcium and phosphate, both of which were recovered in the form of a high molecular weight fetuin-mineral complex [93]–[96]. This mineral complex, or its equivalent prepared from calcium and phosphate added to rat serum, also contained low amounts of matrix γ-carboxyglutamic acid, secreted phosphoprotein-24, and albumin [93]–[96]. Following treatment with etidronate, an increase in the concentration of γ-carboxyglutamic acid was detected, but the level of fetuin-A was decreased by 50% as it was cleared from the body in association with the mineral complexes [94]. In similar studies, decalcified collagen fibrils of rat bones [97] or devitalized aortas [98] were found to re-calcify when incubated in serum under physiological concentrations of calcium and phosphate ions. The re-calcification was attributed to one or more putative bone calcifying factor(s) which were found to be trypsin-sensitive and estimated to have a molecular weight of 55–150 kDa [97], now known to require phosphorylation by alkaline phosphatase in order to become activated [99]. Whether similar protein(s) are involved in the nucleation of HAP associated with the serum granulations and NB seen here is currently unclear.

That fetuin-A is indeed involved in the formation and clearance of the mineral complexes has been elegantly established by observations on fetuin-A knockout mice, shown to spontaneously develop ectopic calcification of soft tissues [91]. In humans, serum fetuin-A deficiency has been linked to calcification and cardiovascular mortality [100]–[103]. From these studies, the lowest concentration of fetuin-A required to prevent spontaneous calcification of calcium phosphate in the body was estimated at 0.2 mg/ml [100], a value close to the minimal concentration of 0.1 mg/ml of fetuin-A estimated to be necessary to stabilize CPP in the presence of other acidic serum proteins [30]. Since fetuin-A is a negative acute phase protein, its expression spontaneously decreases in states of inflammation [104], [105] and this may have an effect on the degree of calcification observed in pathological conditions.

An analysis of the propensity for CPP to form in the body also needs to take into account the amount of albumin and other calcium and apatite binding proteins as well as the calcium phosphate ion product present at the site of calcification [30], [83]. Furthermore, in addition to albumin, several apolipoproteins and complement components have consistently been found in association with serum granulations. It is not clear for example whether these proteins, especially in the case of apolipoproteins, are part of the same protein scaffold or perhaps they may represent distinct particles (lipoprotein particles?) that nonetheless become calcified under the continued presence of excess calcium phosphate. This possibility must be considered in future studies especially since the granulations seen here are clearly heterogeneous from morphological and chemical standpoints. As for the complement components 3 and 4A that have been found to bind so avidly the serum granulations observed here, it is not clear whether they play any immunological or pathophysiological role.

Once formed, the calcium and apatite granulations seen here are probably siphoned for clearance, probably through the RES, as it has also been suggested to be the case for CPP [30], [40], [83]. In the context of CPP, several earlier studies have demonstrated opsonizing properties associated with fetuin-A, which lead to the enhancement of phagocytosis and macropinocytosis by macrophages of apoptotic cells, DNA, and dextran and latex particles [106]–[108]. Intriguingly, the removal of calcified particles as seen in bone remodeling and in kidney clearance is largely non-inflammatory [83]. Thus, even the large deposits seen in fetuin-A knockout mice show no signs of localized inflammation [83]. This anti-inflammatory effect has been attributed tentatively to the influence of polyanions like spermine and anti-inflammatory cytokine proteins like transforming growth factor-β that may become associated with the CPP [reviewed in ref. 83]. On the other hand, the injection of apatite particles has been shown to be fatal to Wistar rats as it induces infarction and acute thrombosis of blood vessels in major organs like the liver and the lungs [109], a finding thought to mimic clinical settings in which unstable atherosclerotic lesions are also known to produce thrombosis and even myocardial infarction. In addition, various materials that have been nano-sized are thought to possess altered, often increased, toxicity on living organisms compared to the same original materials [reviewed in ref. 110], an observation which may be related to the study which found that intravenous injections of apatite nanoparticles produced vascular endothelial damage in rabbits [27]. It is also possible that the main difference between these various experimental set-ups may lie in the extent of protein coating of these particles, presumably with a protective and anti-inflammatory role assigned to the protein layer that is absent in simple mineral phases—an important distinction that warrants more detailed studies.

The potential involvement of other proteins and factors associated with the calcium granulations seen here suggests that these entities are much more complex than what fetuin-A alone can possibly explain. Thus, while the elegant studies performed by Price, Jahnen-Dechent, Heiss, Schäfer, Ketteler, and their colleagues have been centered on the role of fetuin-A as the basis for the formation of mineralo-protein complexes, it appears that the role of other proteins and factors must also be taken into account to explain the biology of the serum-derived calcium granulations observed here.

It appears that the serum calcium granulations seen here are also closely linked to normal bone formation as well as ectopic calcification. Electron microscopy studies have established that the mineral precursor of bone and enamel actually consists of round amorphous nanoparticles of calcium phosphate with small sizes ranging from 5 to 50 nm which take form inside the collagen fibrils and that fuses in a co-linear fashion until filaments are formed [64], [111]–[114]. The precipitation of these round amorphous nanoparticles may be due to the presence of numerous divalent cations able to freely diffuse inside the fibril and inhibit the formation of larger and more crystalline calcium phosphate crystals. This type of mineralization is akin to the formation of “mesocrystals” consisting of superstructures formed from a repetition of individual crystals or “nanobuilding-blocks” [115], [116]. However, the crystalline particles described in the present study as aggregated polycrystalline material do not seem to possess crystallographically-oriented subunits which usually give mesocrystals a much higher degree of crystallinity [116]. Likewise, the calcium-containing nanoparticles or granulations seen here may represent precursors deployed for the purpose of tissue calcification, when needed. It is conceivable that an essentially similar association between calcium and apatite binding proteins and mineral phases, with the resultant formation of spherical nanoparticles, is required for biomineralization.

From a yet another perspective, the calcium and apatite complexes seen here appear to resemble calcium granules found widely in nature [see ref. 29 for a most insightful review]. As such, calcium granules or inclusion bodies appear to be the preferred form of storage or sequestration of calcium in nature [29]. They have been studied in more details in crustaceans [117], arthropods [118], and a variety of invertebrates [119] where they have been found to incorporate other metallic elements, essentially contributing to metal detoxification. In the human blood, calcium granules have been described recently in platelets [43] and have been found to be identical to the so-called “acidocalcisomes,” intracellular deposits of calcium phosphate first found in bacteria and later studied extensively in trypanosomes [120]. Such calcium granules have been shown to play a role in the intracellular storage of calcium and phosphate as well as in the maintenance of the pH and osmolarity of the cell [120]. Other blood-related clusters or complexes include proteons and the so-called proteon-nucleating centers (PNC), representing nanoclusters of proteins or protein fragments with metallic elements with unclear physiological role [121].

Calcium granules have also been found in a number of other mammalian tissues, including cartilage, atherosclerotic lesions, kidney and cancer tissues as well as in association with Randall's plaque [54], [55], [122], [123; see refs. 29 and 124 for pictures of calcium granules obtained from a variety of organisms], [Bibr pone.0005421-Ryall1] [including humans]. Calcium-containing granules similar to the serum granulations and NB were also observed in association with the culture of a human leukemia cell line [125]. When subsequently cultured in 10% FBS in the absence of this particular cell line, the mineral particles grew slowly in culture and were shown to associate with fetuin-A. Thin section of these same particles visualized by TEM showed the presence of round particles with a thick wall, but, overall, no clear biological activity was noted [125]. Based on the widespread prevalence of these calcium granules in nature, Ryall [29] has convincingly articulated a more plausible model viewing them as innocuous deposits of calcium used for the various functions of waste disposal, osmoregulation, excretion of excess ions, ion storage and mobilization, and detoxification.

While there is marked morphological resemblance between the serum granulations observed here and the earlier described calcium granules [29], it should be noted that our serum granulations also display broader pleomorphic tendencies, with particles eventually coalescing to form shapes like spindles and films. It is possible, and perhaps even likely, that in the continued presence of calcium and phosphate ions the calcium granules found throughout nature may show a similar transition to other phases that may also include spindles and films, as mentioned earlier in the context of Randall's plaque observed in the kidney. Thus, in our mind, the particular shapes assumed by calcium granulations may simply reflect their maturation and the balance between the levels seen for protein and other organic calcification inhibitors versus the levels associated with calcium compounds (that is, calcium carbonate or calcium phosphate as well as other related ions). Accordingly, proportionally higher initial levels of protein and organic inhibitors would be seen to favor the spherical shapes, which appear to transition to other elongated, filamentous and membranous shapes as a function of time as well as a result of ionic changes in the environment. Finally, the possibility also exists that our serum granulations may comprise of populations of distinct and heterogeneous entities, especially since proteins with diverse and well-known functions like apolipoproteins and complement components are all present as part of the protein-mineral scaffold.

As for the involvement of these same granules (or NB) in human pathologies, one area that deserves mention is kidney stone and its link with the formation of Randall's papillary plaque [see ref. 29 for a list of related references]. The latter had been proposed by several NB researchers as indicative of a pathogenic involvement for NB [28]. Ryall [29], and several investigators before her [126]–[128], have instead taken the position that the papillary granules constituting Randall's plaque, seen in virtually all adult kidneys examined—diseased or normal—are expressions of *normal*, rather than anomalous or even pathological, calcium metabolism! These same authors had earlier suggested that the same electron-dense granules observed near the basement membranes of collecting ducts and the interstitium showed gradual migration into a subepithelial location in the renal papillae that then form the so-called Randall's plaque [29], [126]–[129]. As such, they are deemed to represent innocent bystanders rather than agents of disease. This view stands in marked contrast to the position expressed by Kajander and Çiftçioğlu, the original discoverers of NB, along with other researchers, who have proposed NB as major causative disease agents for kidney stone formation through their coincidental presence in kidney tissues harboring the kidney stones as well as their direct association with Randall's plaque [1]–[8], [28]. According to the same authors, NB are not only renotropic but also produce morphologies that are identical to the granules seen in Randall's plaque [1]–[8], [28].

Regarding the involvement of NB in the development of kidney stones as well as other disease conditions, it should be noted that our earlier study provided a detailed analysis questioning NB both as living organisms and as pathogenic agents of disease [13]. For instance, our own findings indicate that all previous immunodetection studies used to ascertain the presence of NB in human tissues were based on the erroneous assumption that monoclonal antibodies marketed as specific for NB were actually detecting NB-specific antigens. In fact, all monoclonal antibodies claimed to be specific for NB that we have studied to date have shown to react strongly to both albumin and fetuin-A, not only of the same species, but even across species [13]. More alarmingly, our study showed that these same antigens used to implicate NB in human tissues may have been derived from the FBS used to culture the human tissues under investigation, creating a bizarre and problematic scenario in which *fetal bovine* antigens presumably detected by the same studies may have inadvertently been used to assert the presence of NB in an increasing number of *human* diseases [13].

In further support of this alternative position on NB that questions their proposed involvement in the development of kidney stones via Randall's plaque, it should be pointed out that Ryall's review [29] contains an impressive collection of thin section TEM images of the lamellar granules comprising Randall's plaque (see her Fig. 10). It should immediately be obvious that the granular morphologies seen there are indistinguishable from those observed for calcium granules found throughout nature as well as the serum granulations reported here. In Ryall's words, “It is exceedingly puzzling that nowhere in the large and expanding literature devoted to nanobacteria has there been a single reference to the longstanding, large body of literature attesting to the existence of calcium-containing granules in other organisms—despite the fact that their physical and biochemical characteristics are so similar.” In many respects, our present study vindicates the view that the same calcium granulations found in the serum, deemed to represent remnants of normal calcium and apatite metabolism, are not only morphologically and chemically identical to the putative NB, but are probably also found in all other body fluids and tissues, as well as throughout nature.

The NB field (including the earlier “nannobacteria” literature published on geological specimens) has probably garnered more media attention and controversy than any other field in recent memory [see refs. 130]–[142 for a list of editorials written on NB in both main science journals and popular science magazines, aside from numerous opinions and reviews presented elsewhere]. These controversies have stemmed not only from the extraordinary claims made for NB as exotic and novel microorganisms or as transmissible agents of disease, but also from a lack of biochemical, microbiological, and structural data required for the validation of such claims [see refs. 1]–[8 and the more recent review in ref. 143 advocating the claims made; see also the excellent critical review in ref. 12 questioning the validity of these same claims]. It is this same deficiency of information that we have attempted to address here and elsewhere [13], by focusing on the structural and biochemical characteristics of NB-related entities. Our studies demonstrate a marked similarity between putative NB, serum calcium granulations, and calcium granules found throughout nature, indicating that these entities are closely related, if not identical, and that they probably represent innocuous byproducts and remnants of physiological reactions needed to ensure proper calcium and phosphate homeostasis.

The calcium granulations described in this study also share some characteristics with the so-called matrix vesicles (MV), which represent another kind of calcium phosphate template implicated in the formation of mineralized tissues in the body. Skeletal cells such as osteoblasts, chondroblasts, and odontoblasts control the deposition of minerals in the body by releasing MV which initiate calcification in selected and restricted areas destined to be mineralized [reviewed by Anderson et al. in ref. 144]. MV, consisting of membrane-bound vesicles of small sizes (50–200 nm), have thus been shown to initiate the precipitation of calcium phosphate in bones, cartilage, and teeth [144]. The mechanism of MV calcification apparently involves an increased concentration of calcium and phosphate ions inside MV through the action of calcium transporters like annexins [145] as well as enzymes like alkaline phosphatase [146] and ATPases [147]. These latter enzymes are known to inactivate pyrophosphate and possibly other calcification inhibitors, thereby releasing inorganic phosphate from various phosphate-containing molecules [144]. In addition, MV may contain phospholipids which bind calcium and are thought to play a role in the nucleation of apatite [148]. Upon exposure to high concentrations of calcium and phosphate ions, a calcium phosphate precipitate forms inside MV and is eventually released into the surrounding extracellular matrix following membrane breakdown which is apparently executed by phospholipases [149] as well as by proteases already present in MV [150]. Although MV are usually present only in the body's mineralizing compartments, MV-like structures have been detected in calcified vasculatures and implicated as a possible nucleating agent responsible for the deposition of apatite in these and other ectopic calcification sites [144]. It has also been proposed that apoptotic bodies resembling MV-like structures are released by vascular smooth muscle cells undergoing apoptosis when, for instance, they are confronted with high concentrations of calcium and phosphate [151], [152]. These MV-like formations were shown to harbor fetuin-A, but the presence of this protein abrogated their ability to induce calcification [151], [152]. Presumably, by means of additional calcium phosphate binding and saturation, these same MV-like formations may become seeds for further apatite deposition, in line with the dual inhibition-seeding concept for granule formation demonstrated here. Other vesicles present in the blood have been described under various names like membrane microparticles, shed membrane particles, membrane vesicles, extracellular vesicles, exosomes, among others [153]. Although these different vesicles were implicated mainly in intercellular communication, hemostasis, and immunity [153], it remains unclear whether such MV-like structures or the MV described earlier are related to the serum granulations seen in this study. Our findings of several apolipoproteins associated with the scaffold of serum granulations studied here as well as preliminary data pointing to the presence of lipids in association with NB-like particles [13] would suggest a role for lipids in the assembly of both serum granulations and NB. Further studies will be needed to ascertain whether these are related in anyway to the MV described earlier. However, given that the sera used here to prepare the serum granulations or to culture NB were obtained from healthy human volunteers or from commercially available FBS, it appears unlikely that such material contains MV in sufficient amounts to drive the formation of the mineral formations observed. This is not to say that the two pathways may not in fact coexist or even overlap in pathological calcifications of the vasculature exposed simultaneously to blood-related as well as extracellular influences. More experiments are clearly needed to address these questions.

An analogy can also be drawn between the formation of the calcium granulations presented here and the formation of polymer gels from dissolved organic matter as described earlier [154]. In the latter study, filtration of ocean water through 0.22 µm filters yielded colloid particles with diameters between 2 to 200 nm which, upon incubation at 20°C for several hours, gradually increased in size (200 nm to 1 µm), culminating in the formation of a network of organic matter. The network or matrix which readily trapped carbohydrates, proteins, and lipids from the sea water was also found to contain calcium carbonate crystals [154]. The crystallization of calcium carbonate inside this network was attributed to a Donnan effect which predicts that the concentration of calcium within the matrix should be higher than that of the exterior due to the polyanionic nature of the matrix [154]. When the pH of the water slightly increased, precipitation of calcium carbonate occurred within the matrix while the outside water remained essentially free of crystals [154]. Based on these findings, it would be interesting to verify whether the factors found to influence the formation of polymer gels would have a similar effect on the formation of calcium and apatite granulations prepared from serum.

Finally, it should be noted that similar granulations have been observed in human blood specimens and likewise described over the past century as “inclusion bodies,” “filterable bodies,” “microzymas,” “Granuligera,” “protids,” “somatids,” “blood bacteria,” among others [155], [156], but that have long been relegated to questionable, if not spurious, status by the mainstream microbiological establishment. Among the remarkable features that these entities share with each other as well as with serum calcium granulations and NB are the small, sub-micrometer sizes granting them membrane-filterability, their proliferation like bacteria, and their marked pleomorphism. With respect to pleomorphism, it is intriguing that complex biological cycles representing multiple forms ranging from round and bacilli-shaped bacteria to filamentous fungus- and yeast-like projections have been proposed for each one of these entities [Bibr pone.0005421-Wainwright1] [see citations contained in refs. 155 and 156]. Our results suggest for the first time that the calcium and apatite granulations reported here may provide an alternative explanation and basis for some, if not all, aspects of this phenomenology.

## Materials and Methods

### Preparation of serum pellets

Human blood was collected from healthy human volunteers using a conventional venipuncture method. The use of human samples was approved by the Institutional Review Board of Chang Gung Memorial Hospital (Gueishan, Taiwan, Republic of China) and supported by written informed consents. Whole blood was collected into Vacutainer tubes without anticoagulant (Becton, Dickinson & Company, Sparks, MD, USA) and centrifuged at 1,500×*g* for 15 min at room temperature. The supernatant corresponding to HS was saved into another tube. FBS (Biological Industries, Kibbutz Beit Haemek, Israel) and HS were successively filtered through 0.2 µm and 0.1 µm membranes prior to use. Serum pellets were prepared by adding sterile solutions of either 0.25 M CaCl_2_, pH 7.4, or 0.25 M Na_2_HPO_4_ (adjusted to pH 7.4 with 0.25 M NaH_2_PO_4_), or a combination of both, into filtered FBS or HS (2.5 ml) at final concentrations ranging from 12 mM to 48 mM for CaCl_2_, 6 mM to 24 mM for Na_2_HPO_4_, or 1 mM to 2 mM of each when both CaCl_2_ and Na_2_HPO_4_ were added. The serum pellets shown in Figs. 1, 2 and 5–11 were prepared either from untreated serum (labeled in the figures as FBS-1/HS-1) or by adding either 48 mM CaCl_2_ (labeled as FBS-2/HS-2), 24 mM Na_2_HPO_4_ (labeled as FBS-3/HS-3), or 2 mM of both CaCl_2_ and Na_2_HPO_4_ (labeled as FBS-4/HS-4) to the serum. Ion solutions were added drop-wise with vigorous agitation to avoid precipitation. Treated or control sera were incubated at 37°C for 2 hours (used for Figs. 3 and 4) or overnight at room temperature (used for Figs. 1, 2, and 5–11) with gentle, end-over-end shaking. Following incubation, the serum solutions were centrifuged at 16,000×*g* for 1 hour and the pellets were washed successively with 0.2 µm membrane-filtered Dulbecco's Modified Eagle's Medium (DMEM; Gibco, Carlsbad, CA, USA) or HEPES buffer (20 mM HEPES, 1 mM CaCl_2_, 2 mM Na_2_HPO_4_, 0.02% sodium azide, and 0.15 M NaCl, pH 7.4) using the same centrifugation steps.

Alternatively, the untreated serum pellets were prepared by incubating filtered FBS or HS (38 ml) with gentle agitation overnight at room temperature, followed by centrifugation at 140,000×*g* at 4°C for 2 hours using a L8-80M ultracentrifuge (Beckman Coulter, Fullerton, CA, USA). The pellets were then washed twice with either filtered DMEM, HEPES buffer, or double distilled water using the same centrifugation steps.

For Fig. 1, the pellets were resuspended in 100 µl of HEPES buffer. Small aliquots of these suspensions (2 µl to 15 µl) were diluted into serum-free DMEM, DMEM without calcium (Gibco), or DMEM without phosphate (Gibco) in a final volume of 1 ml using 24 well plates. 1 ml of serum-free DMEM receiving no inoculation was also incubated in the same conditions as a negative control. The plates were incubated at 37°C in cell culture conditions. Visual inspection, photography, and optical density monitoring at A_650_ were done as before [13]. Serum pellets described as FBS-2′ and FBS-2″ in Fig. 7 were prepared by adding 48 mM CaCl_2_ to FBS, followed by either a short incubation of 1 hour (FBS-2′) or overnight (FBS-2″) at room temperature, and were processed as described above.

### Culture and preparation of NB specimens

Putative NB were obtained from FBS or HS as described earlier [2], [157]. Briefly, FBS or HS were filtered through a 0.2 µm membrane, diluted 1∶10 to 1∶300 in DMEM, followed by incubation at 37°C in cell culture conditions for several weeks. The *Nanobacterium* sp. strains designated as DSM 5820 and DSM 5821, initially isolated from commercially available FBS [36], was obtained from the German Collection of Microorganisms and Cell Cultures (DSMZ; Braunschweig, Germany). This strain, along with two others used in our previous study [13], had earlier been deposited by Dr. E. Olavi Kajander (Nanobac Oy, Neulaniementie, Finland) into the DSMZ in association with a patent issued in 1992 describing the isolation of NB (USA patent #5,135,851). The strain of NB designated as “nanons” [15] was provided by Dr. Didier Raoult (Unité des Rickettsies, Centre National de la Recherche Scientifique UMR 6020, Faculté de Médecine, Marseille, France). This strain was initially isolated from FBS and named “*Nanobacterium* sp. strain Seralab 901045” [15], [157]. The different NB strains were scraped from a T-75 cm^2^ flask (Corning, Inc., Corning, NY, USA) showing abundant culture and an aliquot of 2 ml was centrifugated at 16,000×*g* at room temperature for 15 min. The pellet was washed twice with HEPES buffer or double distilled water using the same centrifugation steps. NB were then resuspended in double distilled water and used as such for the different microscopy, spectroscopy, and proteomic analyses.

### Preparation of seeding-pellets obtained from serum treated with trypsin or chymotrypsin

Serum pellets used for the experiment described on Fig. 12 were prepared by adding aliquots of a 0.2 µm filtrated solution of 5% (w/v) porcine pancreas trypsin (Sigma, St-Louis, MO, USA) or bovine pancreas chymotrypsin (Sigma) into either filtrated FBS or HS (1 ml) to a final concentration of 0.5% (v/v). The solutions were then incubated with gentle end-over-end agitation at 37°C for 2 hours or overnight. After incubation, the solutions were centrifuged at 16,000×*g* at room temperature for 1 hour. The pellets were then washed twice with HEPES buffer and were resuspended in 100 µl of the same buffer. Small aliquots (10 µl) of the resuspended pellets were inoculated into DMEM and incubated in cell culture conditions. FBS and HS that received no treatment with trypsin or chymotrypsin were processed exactly the same way as controls.

### Optical dark field microscopy

Washed serum pellets and NB samples were resuspended in double distilled water and aliquots were deposited on glass slides. The samples were visualized without fixation or staining with a BX-51 optical microscope (Olympus, Tokyo, Japan) equipped with a dark field condenser (Cerbe Distribution, Inc., Sherbrooke, Québec, Canada) and a 100× oil immersion objective with iris (UPlanFLN; Olympus). The specimens were visualized at a magnification of 1,000× and the images were acquired with a Spot Flex color, charge-coupled device (CCD) camera (Diagnostic Instruments, Inc., Sterling Heights, MI, USA). Commercially available HAP (buffered aqueous suspension, 25% w/v; Sigma) was used here as well as throughout this study as a control.

### Electron microscopy

For scanning electron microscopy (SEM), washed serum pellets and NB specimens were resuspended in double distilled water and deposited on formvar carbon-coated grids. After drying overnight under a laminar flow hood, the specimens were coated with gold for 90 seconds and observed with a SEM S-5000 field-emission scanning electron microscope (Hitachi Science Systems, Tokyo, Japan). For transmission electron microscopy (TEM), washed serum pellets and NB samples were deposited on formvar carbon-coated grids without staining; the excess liquid was removed with an absorbent paper, and the grids were then dried overnight. For thin sections, the same specimens were washed twice in double distilled water and dehydrated with two washes of 100% ethanol. The samples were then embedded in Epon 812 resin (Electron Microscopy Sciences, Fort Washington, PA, USA) and incubated at 72°C for 2 days to allow resin polymerization. Thin sections were cut using a Leica Ultracut UCT microtome (Leica Microsystems GmbH, Wetzlar, Germany) and were transferred on formvar carbon-coated grids. Specimens prepared for TEM were observed without staining with a JEOL JEM-1230 transmission electron microscope (JEOL, Tokyo, Japan) operated at 120 keV.

### X-ray and electron diffraction analyses

Washed serum pellets and NB specimens resuspended in double distilled water were deposited on glass slides and dried overnight. Powder X-ray diffraction spectroscopy (XRD) was performed using a Bruker AXS D5005 X-ray diffractometer (Bruker AXS, Madison, WI, USA) equipped with a sealed 2.2 kW copper X-ray source, a diffracted beam monochromator, and a scintillation counter detector. Experimental diffraction spectra were compared with the database of the Joint Committee on Powder Diffraction and Standards (JPCDS) in order to identify the chemical formula of each crystalline compound. Electron diffraction patterns were acquired with a JEOL JEM-1230 transmission electron microscope (JEOL) operated at 120 keV.

### Energy-dispersive X-ray spectroscopy

Aliquots of washed serum pellets and NB specimens resuspended in water were deposited on formvar carbon-coated grids and dried overnight. Energy-dispersive X-ray spectroscopy (EDX) was performed using a SEM S-3000N scanning electron microscope (Hitachi Science Systems) equipped with an EMAX Energy EX-400 EDX device (Horiba, Tokyo, Japan) as described before [13]. Commercially available CaCO_3_ (A.C.S. grade reagent, purity 99.6%, Mallinckrodt Baker, Inc., Phillipsburg, NJ, USA) and calcium phosphate tribasic (Kanto Chemical Co., Tokyo, Japan), also labeled as Ca_3_(PO_4_)_2_ in the figures, were used for comparison with the experimental specimens.

### Fourier-transformed infrared spectroscopy

Washed and dried serum pellets and NB specimens were mixed 1∶100 (w/w) with KBr powder and compressed with a hand press to form a thin pellicle. FTIR spectroscopy was performed using a Nicolet 5700 FTIR spectrometer (Thermo Fisher Scientific, Waltham, MA, USA) equipped with a deuterated triglycine sulfate (DTGS) detector. Each spectrum was obtained at a resolution of 4 cm^−1^ and represented an average of 32 consecutive scans.

### Micro-Raman spectroscopy

Washed serum pellets and NB samples were resuspended in double distilled water and deposited on glass slides. The specimens were then dried overnight at room temperature under a laminar flow hood. Micro-Raman spectroscopy was performed using the inVia Raman confocal microscope (Renishaw, Stonehouse, UK) equipped with a CCD detector. A laser beam of 633 nm operated at 17 mW was focused on the specimens and provided the excitation source.

### Sodium dodecyl sulfate polyacrylamide gel electrophoresis (SDS-PAGE)

2 µl aliquots of each 100 µl pellet suspension described earlier were successively mixed with 14 µl of 50 mM EDTA and 4 µl of concentrated “loading buffer” (0.313 M Tris-HCl pH 6.8, 10% SDS, 0.05% bromophenol blue, 50% glycerol, 12.5% β-mercaptoethanol). The protein samples were heated at 95°C for 5 min prior to gel electrophoresis using a mini-gel system (Hoefer, Holliston, MA, USA). The gels were then stained with Coomassie blue as described [13].

### Matrix-assisted laser desorption ionization-time of flight (MALDI-TOF) mass spectrometry

Protein bands from gels were excised, reduced and alkylated, in-gel digested with trypsin, and further processed for mass spectrometry (MS) [13]. MS and tandem MS/MS spectra were obtained with an Ultraflex TOF-TOF mass spectrometer (Bruker Daltonics, Bremen, Germany). Selected peaks were fragmented by the LIFT method to confirm the identity of the highest score protein by MS/MS analysis.

In addition, serum pellet and NB samples were submitted to in-solution trypsin digestion as described earlier [13]. Serum pellets and NB washed twice in DMEM and once in distilled water were successively reduced, alkylated, and digested with trypsin directly in solution [13]. Peptides were dried in a vacuum centrifuge at room temperature for 10 min. The dried peptides were then resuspended in 0.1% formic acid (Sigma), loaded onto a reversed-phase liquid chromatography trap column (Zorbax 300SB-C18, Agilent Technologies, Wilmington, DE, USA), and separated using a 10 cm analytical C_18_ column (New Objective, Woburn, MA, USA). Elution was performed with a succession of different solutions containing an increasing concentration of formic acid (starting with 0.1% formic acid diluted in 99.9% acetonitrile). The liquid chromatography column was placed in line with a 2-D linear ion trap mass spectrometer (LTQ-Orbitrap; Thermo Fisher) operated using the Xcalibur 2.0 software (Thermo Fisher).

Mass fingerprints were searched against the Swiss-Prot database using the MASCOT search engine (Matrix Sciences, London, UK) as described before [13]. The criteria for positive protein identification included a score above the MASCOT threshold of chance expectation value (p<0.05), a molecular weight corresponding to the band obtained on the SDS-PAGE, and a confirmation of the highest score protein by MS/MS analysis of two selected peaks.
